# SANCDB: an update on South African natural compounds and their readily available analogs

**DOI:** 10.1186/s13321-021-00514-2

**Published:** 2021-05-05

**Authors:** Bakary N’tji Diallo, Michael Glenister, Thommas M. Musyoka, Kevin Lobb, Özlem Tastan Bishop

**Affiliations:** 1 Research Unit in Bioinformatics (RUBi), Department of Biochemistry and Microbiology, Rhodes University, Makhanda/Grahamstown, 6140 South Africa; 2 Department of Chemistry, Rhodes University, Makhanda/Grahamstown, 6140 South Africa

**Keywords:** SANCDB, Natural products, Commercial analogs, Drug discovery

## Abstract

**Background:**

South African Natural Compounds Database (SANCDB; https://sancdb.rubi.ru.ac.za/) is the sole and a fully referenced database of natural chemical compounds of South African biodiversity. It is freely available, and since its inception in 2015, the database has become an important resource to several studies. Its content has been: used as training data for machine learning models; incorporated to larger databases; and utilized in drug discovery studies for hit identifications.

**Description:**

Here, we report the updated version of SANCDB. The new version includes 412 additional compounds that have been reported since 2015, giving a total of 1012 compounds in the database. Further, although natural products (NPs) are an important source of unique scaffolds, they have a major drawback due to their complex structure resulting in low synthetic feasibility in the laboratory. With this in mind, SANCDB is, now, updated to provide direct links to commercially available analogs from two major chemical databases namely Mcule and MolPort. To our knowledge, this feature is not available in other NP databases. Additionally, for easier access to information by users, the database and website interface were updated. The compounds are now downloadable in many different chemical formats.

**Conclusions:**

The drug discovery process relies heavily on NPs due to their unique chemical organization. This has inspired the establishment of numerous NP chemical databases. With the emergence of newer chemoinformatic technologies, existing chemical databases require constant updates to facilitate information accessibility and integration by users. Besides increasing the NPs compound content, the updated SANCDB allows users to access the individual compounds (if available) or their analogs from commercial databases seamlessly.

**Graphic abstract:**

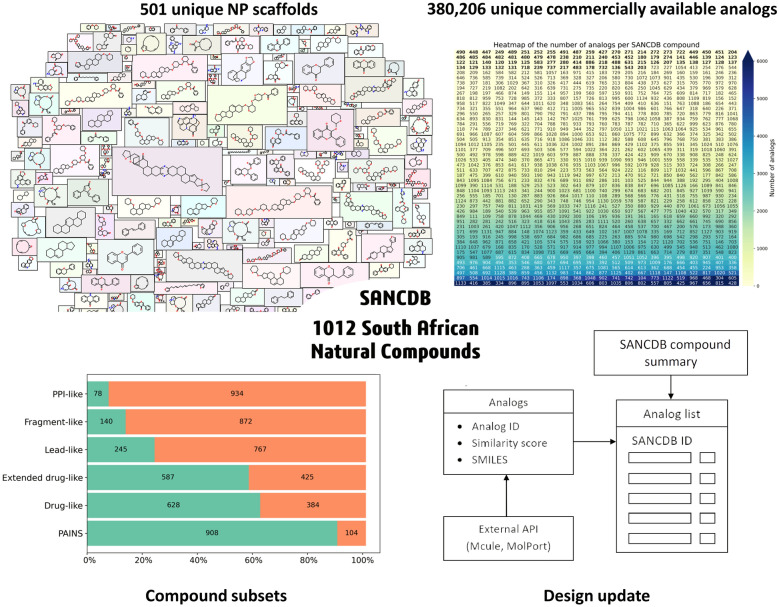

**Supplementary Information:**

The online version contains supplementary material available at 10.1186/s13321-021-00514-2.

## Introduction

Throughout history, natural products (NPs) have benefited mankind in food, pesticides, cosmetic products and as drugs [1]. NP research has, especially, been a growing field in modern drug discovery as they offer unique chemical scaffolds [2], hence a greater structural diversity than synthetic ones, and they cover a large area of chemical space [3]. Between 2010 and 2019, NPs contributed to 25–33% of approved small molecules [4], and it is estimated that they represent 35% of medicines [5]. Some of the notable approved drugs, either from pure or derived NPs, include lefamulin, the aminoglycoside antibiotic plazomicin; tafenoquine succinate, an antimalarial agent; and aplidine, an anticancer agent [4]. Given the high interest in NP research, over 120 NP databases and collections have been developed, and are continuously being updated [1, 3] with new information and functionalities.

The South African Natural Compounds Database (SANCDB; https://sancdb.rubi.ru.ac.za/) is a fully referenced database of NPs derived from sources within South Africa [6]. The database and website were established by the Research Unit in Bioinformatics (RUBi) in 2015. The main content of the database is a set of NP chemical structures in different chemical formats linked to their primary literature reference. Since its creation, the database has attracted significant interest in diverse domains including NP research, drug discovery, cheminformatics and machine learning with up to 52 citations. Besides its use in hit identification in drug discovery studies, SANCDB content has also served as training data for machine learning models [7, 8]; training data for a NPs likeness scorer (NaPLeS) [7]; and for NP-Scout, a machine learning approach for the identification of NPs [9]. Similarly, the database compounds have served to train STarFish, a target fishing model for NPs [8]. SANCDB was also utilized as an intermediate node for data integration into larger or more specialized information systems. An example is Natural Product Activity and Species Source (NPASS), which is a NP activity and species source database built on some NPs data resources, including SANCDB [10]. Similarly, SANCDB data has also been used in the COCONUT (COlleCtion of Open Natural ProdUcTs) database [11].

The usage of the South African natural compounds as drugs is yet to be reported. However, the search for potential hits with activity against infectious agents and cancer is on the rise, and may lead to the identification of drugs in the near future [12]. In terms of drug discovery studies, the database has been used for identification of hit compounds against the active (orthosteric) site of various biological drug targets in diseases including malaria, trypanosomiasis and severe acute respiratory syndrome Corona Virus 2 (SARS-COV-2) [13–18]. Additionally, potential allosteric modulators such as 20(29)-lupene-3β-isoferulate (SANC00518) for human Hsp90α [14]; discorhabdin N (SANC00132), for human Hsp72 and Hsc70 [15]; gordonoside A (SANC00456) for *Plasmodium falciparum* Prolyl tRNA synthetase [19] have also been identified from SANCDB.

Here, we present an updated version of SANCDB with a number of new features. Firstly, over the last five years, since the inception of SANCDB, considerable NP research has been performed in the country. For instance, in 2019, Fantoukh et al. isolated 11 compounds from *Aspalathus linearis* [20], and Awolola et al. reported four compounds from the genus *Ficus* [21]. A variety of flavonoids, proanthocyanidins, ellagitannins, oligosaccharides and quinic acid derivatives were isolated from *Myrothamnus flabellifolia Welw* in 2016 [22]. Thus, the updated SANCDB has curated such continuously growing information. Secondly, in the updated version of the database, we provide links to commercially available analogs for each compound to increase the search space and the availability of physical compounds. We hope that this unique feature of SANCDB will later be implemented by other NPs databases. Most of the time NPs’ physical availability is either limited [1] or very expensive due to required isolation methods [5, 23]; and it has been well demonstrated that they can serve as good start points for the development of synthesizable analogs which may lead to effective drugs [24]. Finally, we include a scaffold analysis for all compound entries; this was undertaken to determine the database chemical diversity, which is an important aspect in the exploration of potential hit compounds [25]. Compound classification in SANCDB is also revisited.

## Methods

### Update of the database

NPs isolated from South African sources were searched in the literature, and uploaded through the earlier pipeline described in the preceding publication [6]. Elsevier's abstract and citation database (Scopus) Application Programming Interface (API) was used to identify more references. This allowed access to the scholarly databases indexed by Scopus [26] for collection, parsing and extraction of organized literature references. From the current set of references in SANCDB, we retrieved the list of all authors using the reference Digital Object Identifier (DOI). Using the Scopus API [26], a list of all publications associated with each author was retrieved. Both redundant publications and those in which none of the authors had a South African affiliation were removed as a pre-filtering step. From the remaining list of publications, the abstracts, and if available, the full text of the articles, were retrieved. Keywords “South Africa”, “compound” and “isolate” were then searched in the abstracts and full texts, removing documents not presenting any of these keywords. The resulting list of publications was then searched in SciFinder [27] to find each compound’s Chemical Abstracts Service (CAS) [27] number. References were then checked to confirm that sources were indeed from South Africa. Using an updated pipeline described in the original publication [6], new compounds were uploaded into the database. Compound sources were mapped to their genera, families and kingdoms using pygbif [28] a Python client for the Global Biodiversity Information Facility (GBIF) [29] API. PubChemPy [30] was used to retrieve additional information on chemical compounds utilizing their CAS number as query. Compound IDs for different databases (ChEMBL [31], DrugBank [32], ZINC [33], PubChem [34]) were automatically retrieved from PubChem [34]. Besides the previously assigned compound classification generated manually, additional automated classification based on ClassyFire [35] was also included. To allow diverse usage of the compounds in docking studies, structures were prepared in different ready-to-dock formats, viz AutoDock *pdbqt* and Schrödinger Maestro format [36]. All aromatic compound structure depictions were standardized to the Kekulé form.

### Analogs

Analogs for each SANCDB entry were extracted from the Mcule [37] and Molport [38] databases. The set of purchasable compounds in SMILES format from each of these databases (Version October 2019, latest version at that date) was downloaded. Similarity scores (Tanimoto coefficient) were computed using OpenBabel (Version 2.3) [39] fingerprint FP2 [40], a path-based fingerprint which indexes compounds linear fragments up to seven atoms. With the set of indexed fragments, a hash number from zero to 1020 was used to set a bit in a 1024-bit vector. A Tanimoto coefficient of 0.6 or greater was used as cut-off for analog identification. These steps have been incorporated into an automated update pipeline in the backend which fetches updated analog data from the respective Mcule and MolPort database APIs monthly. The front end of the database has also received updates and additions to integrate information about the newly added compounds and analogs.

### Chemoinformatic analysis

SANCDB compounds’ scaffolds were calculated through the Bemis-Murcko decomposition of molecules [3, 25, 41–45]. Scopy [46] was utilized for scaffold calculations. Unique scaffolds from each molecule were assembled with their respective frequencies to generate the molecule cloud [46, 47].

An analysis of compound distributions into drug-like, extended drug-like, lead-like, fragment-like, protein–protein inhibitor-like (PPI-like) subsets was performed (Table 1), using conditions defined previously [48].

**Table 1:** Molecular properties and conditions used to determine compound subsets

Molecular properties	Conditions
Drug-like	MW ≤ 500 & MW ≥ 150 & logP ≤ 5 & nHD ≤ 5 & nHA ≤ 10
Extended drug-like	Druglike & nRot ≤ 7 & TPSA < 150
Lead-like	MW ≥ 250 & MW ≤ 350 & nRot ≤ 7 & logP ≤ 3.5
Fragment-like	nHA ≥ 3 & MW ≤ 300 & nHD ≤ 3 & logP ≤ 3
PPI-like	nRing ≥ 4 & MW > 400 & nHA > 4 & logP > 4

*MW*  Molecular weight, *logP* lipophilicity, *nHA*  number of hydrogen bond acceptor, *nHD*  number of hydrogen bond donor, *TPSA*  total polar surface area, *nRot* number of rotatable bonds and *nRing* number of rings

To assess the coverage of SANCDB chemical space by the analogs, Principal Component Analysis (PCA) and t-Distributed Stochastic Neighbor Embedding (t-SNE) were applied on compounds’ non-normalized Molecular Quantum Numbers (MQN) [49] for dimensionality reduction. MQN descriptors were computed using rdkit.Chem.rdMolDescriptors module of RDKit [50]. T-SNE is a variant of Stochastic Neighbor Embedding calculating similarity between two points in the low-dimensional space using a Student-t distribution [51]. The method has been used for chemical space analysis [52–54]. PCA and t-SNE implementations in scikit-learn were used [55, 56]. A learning rate of 100 and perplexity of 50 were used for t-SNE. Other parameters were kept to default. The 42 dimensions in MQN property space were reduced to two dimensions with both methods. The 3D structures of the identified analogs were prepared using OpenBabel [39] and resulting geometries minimized in RDKit [50] using the Merck Molecular Force Field (MMFF94) [57]. Moreover, the AutoDock ready to dock files ‘*pdbqt*’ were prepared using AutoDock 4 utilities [58]. The data was analyzed in the Jupyter Notebook [59] environment using the Python module pandas [60, 61] and pandas-profiling [62]. The descriptive statistics and plots were done in the same environment.

## Results and discussions

### Database design and website interface

SANCDB uses a MySQL database, managed by the Django framework as described previously [6]. The appearance of the site has seen numerous minor changes such as removing clutter from page elements and menus, using Bootstrap to standardize elements, and adjusting text sizes to give the site a more modern and neater appearance. Redundant and unused JavaScript and CSS libraries have been removed. This reduced the initial page load by 0.5 MB and improved maintainability of the SANCDB codebase.

A similarity search was added to the database search functionalities. The database originally only had a substructure search function. The user can now do a similarity or a substructure search. The query is searched in the database using Open Babel FP2 fingerprints and all compounds having at least 0.6 Tanimoto similarity are returned.

### Compound summary page and analogs

**Fig. 1:**
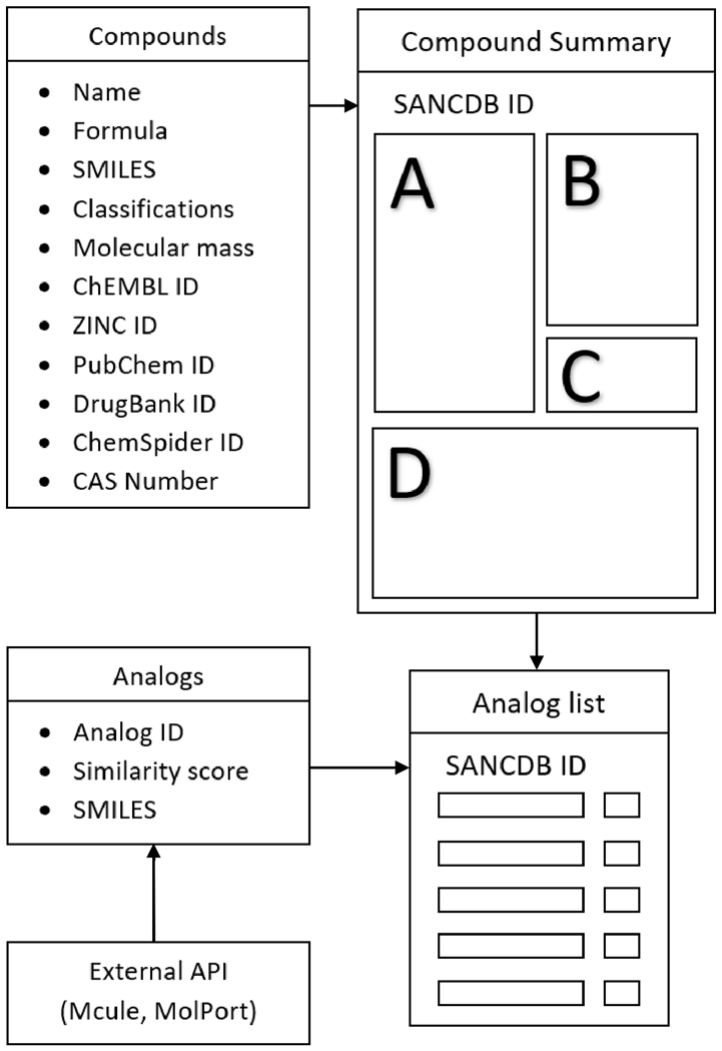
Database schema detailing additions to the existing SANCDB database, compound summary and analog pages. **a** Basic compound information (Name, Formula, SMILES…). **b** Allows users to view 2D and 3D depictions of the compound. **c** Structure download options and navigation to page listing commercially available analogs. **d** References, classifications, other names, sources organisms and uses. Analogs are updated monthly using an automated pipeline to access external database APIs, calculate similarity scores and insert into the local SQL database

As shown in the database schema in Fig. 1, updates featuring the molecular mass and additional compound identification parameters were implemented within the compounds table. Further, a new table was created to link analogs to the respective compounds. Each compound now has the option to view and download commercially available analogs. A total of 380,206 commercially available analogs were added from the Mcule [37] and MolPort [38] databases (at the time of writing). A user can see a list of analogs ordered by similarity score for any compound in SANCDB. Each analog links to its respective entry on the vendor’s website.

From the compound summary page, for each compound there are also links to ChEMBL, DrugBank, PubChem, and ZINC databases, if available. Data points for molecular mass and, when available, a link to ChemSpider have been added to the interface. Standardized compound classifications according to ClassyFire, are displayed in addition to all existing classifications. Previously, compound structures for MOL2, PDB, minimized PDB, SDF and SMILES were available. Subsequently, Maestro and PDBQT files have been added to these options for existing and new compounds.

### Database content

**Fig. 2:**
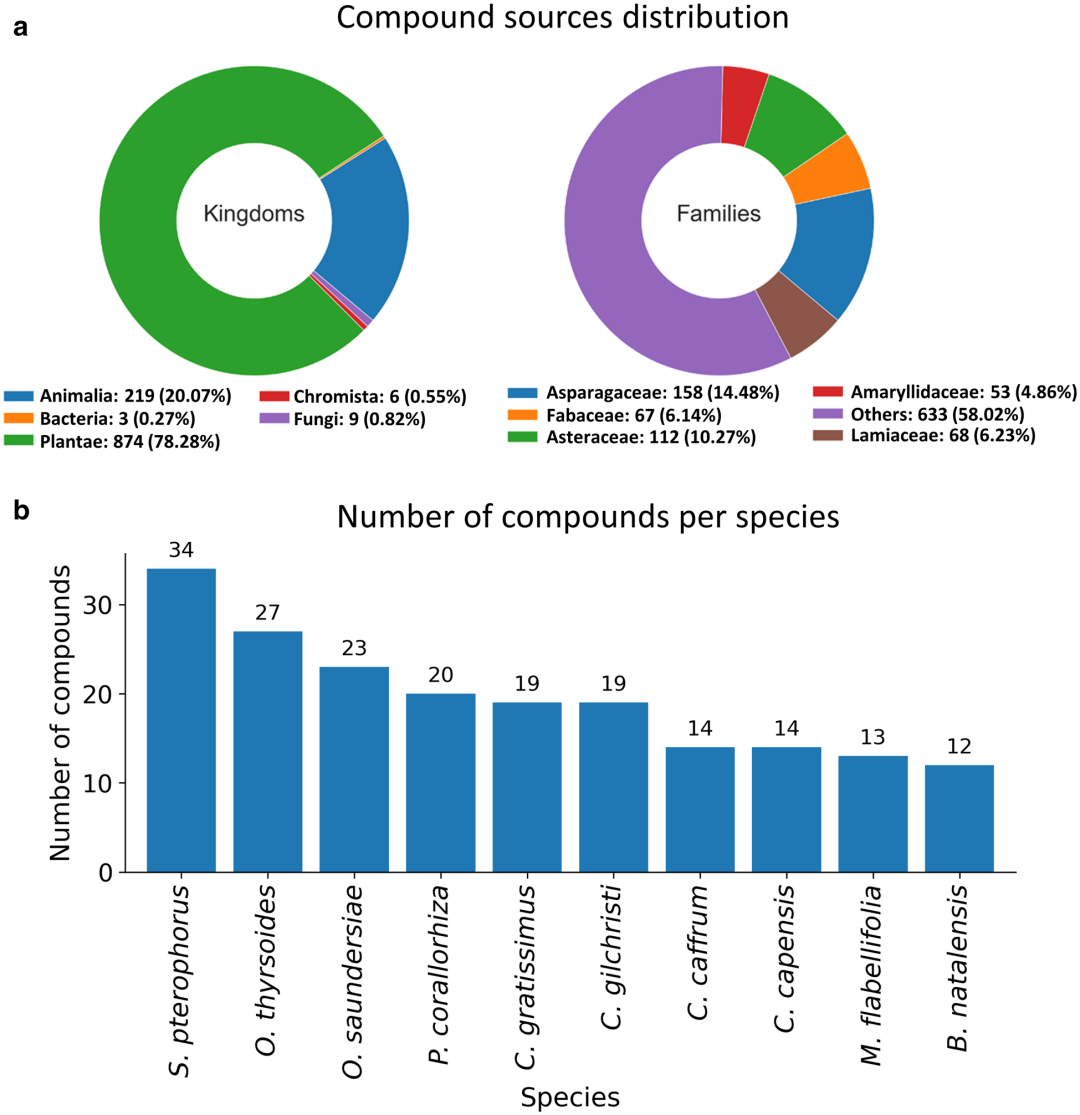
**a** Compound sources distribution. Sources’ species were mapped to their kingdoms, families and genera using pygbif [28]. **b** Most reported species within SANCDB. Only the top 10 species producing highest numbers of NPs are reported

The database initially contained 600 compounds updated here to 1012 compounds from 359 literature references comprising mainly journal articles. Cumulatively, compounds had been isolated from 321 sources, distributed among five biological kingdoms (fungi, bacteria, plants etc.), 104 distinct families and 187 genera. The distribution of the sources of compounds according to their families and kingdoms is shown in Fig. 2a. Plants represented the majority of the sources counting for 854 compounds (78.3%) followed by animals 219 (20.1%), fungi 9 (0.8%), chromista 6 (0.5%) and finally bacteria with 3 compounds (0.3%). This distribution is similar to some of the NPs databases where plants are the primary sources [48]. It is interesting to note the low proportion of compounds, isolated from bacterial and from microbial sources, given that they are major sources of NPs [3]. *Streptomyces sp.* with three isoflavones was the only bacteria species source reported in the entire database. *Clathrina aff reticulum*, *Eurotium rubrum*, *Termitomyces microcarpus* and *Fusarium proliferatum* were the only four fungi. Yet, microbes remain sources for major classes of antibiotics [3]. This low microbial source proportion was also consistent with other NPs databases [48, 63], and some databases only focus on plants [64–66] probably because of their abundance as NP sources. The potential of NPs from microbes in South Africa may be under-explored. A similar observation was made in the source distribution of the Brazilian compound database NuBBE [48]. Plants were found to be the major producer of NPs compared to other sources (animals, fungi, bacteria) [67]. This may be explained by plant uses being more documented in traditional medicines and having easier accessibility than other sources.

Regarding families, Asparagaceae (158–14.48%), Asteraceae (112–10.27%), Lamiaceae (68–6.23%), Fabaceae (67–6.14%) and Amaryllidaceae (53–4.86%) were the top family sources. Regarding genera, *Ornithogalum* (62–5.7%), *Senecio* (58–5.3%), *Eucomis* (39–3.6%), *Salvia* (39–3.6%) and *Plocamium* (38–3.5%) were the top ones.

The top five species with the highest number of isolated compounds were *Senecio pterophorus* (34–3.1%), *Ornithogalum thyrsoides* (27–2.4%), *Ornithogalum saundersiae* (23–2.1%), *Plocamium corallorhiza* (20–1.8%) and *Cephalodiscus gilchristi* (19–1.7%) as shown in Fig. 2b. *Seneccio pterophorus*, is an Asteraceae producing macrocyclic diester pyrrolizidine alkaloids known to be hepatotoxic, carcinogenic, genotoxic and teratogenic [68]. Two species of Ornithogalum plant from the Asparagaceae family: *thyrsoides* (27 compounds) and *saundersiae* (23 compounds) were the second and third sources with the highest number of isolated compounds, respectively. The *thyrsoides* species is widespread in South Africa, Western Cape and known for its cytotoxicity against HL-60 human promyelocytic leukemia cells [69, 70]. *Ornithogalum saundersiae* is an ornamental flower from Mpumalanga, KwaZulu-Natal, and Swaziland, toxic to cattle [71, 72]. Also known as the tube worm [73], *Cephalodiscus gilchristi* is a cephalodiscidae containing highly potent alkaloids against lymphocytic leukemia [74]. This marine invertebrate also produces cephalostatin 1, a potent cell growth-inhibiting compound [73]. Finally, *Plocamium corallorhiza* is a red algae in the family Plocamiaceae, abundant in South Africa known for its halogenated monoterpenes [75, 76]. A common characteristic among these top sources is that they are naturally widespread. Thus, they may be more accessible for compound isolation and extraction studies and the source of more compounds as a result. Information in the database may contribute to biodiversity conservation. Indeed, the above information may contribute to finding NPs source hotspots that conservation efforts may prioritize. None of these species was found in the South African National Biodiversity Institute (SANBI) list of endangered species [77].

### Compounds classification

**Fig. 3:**
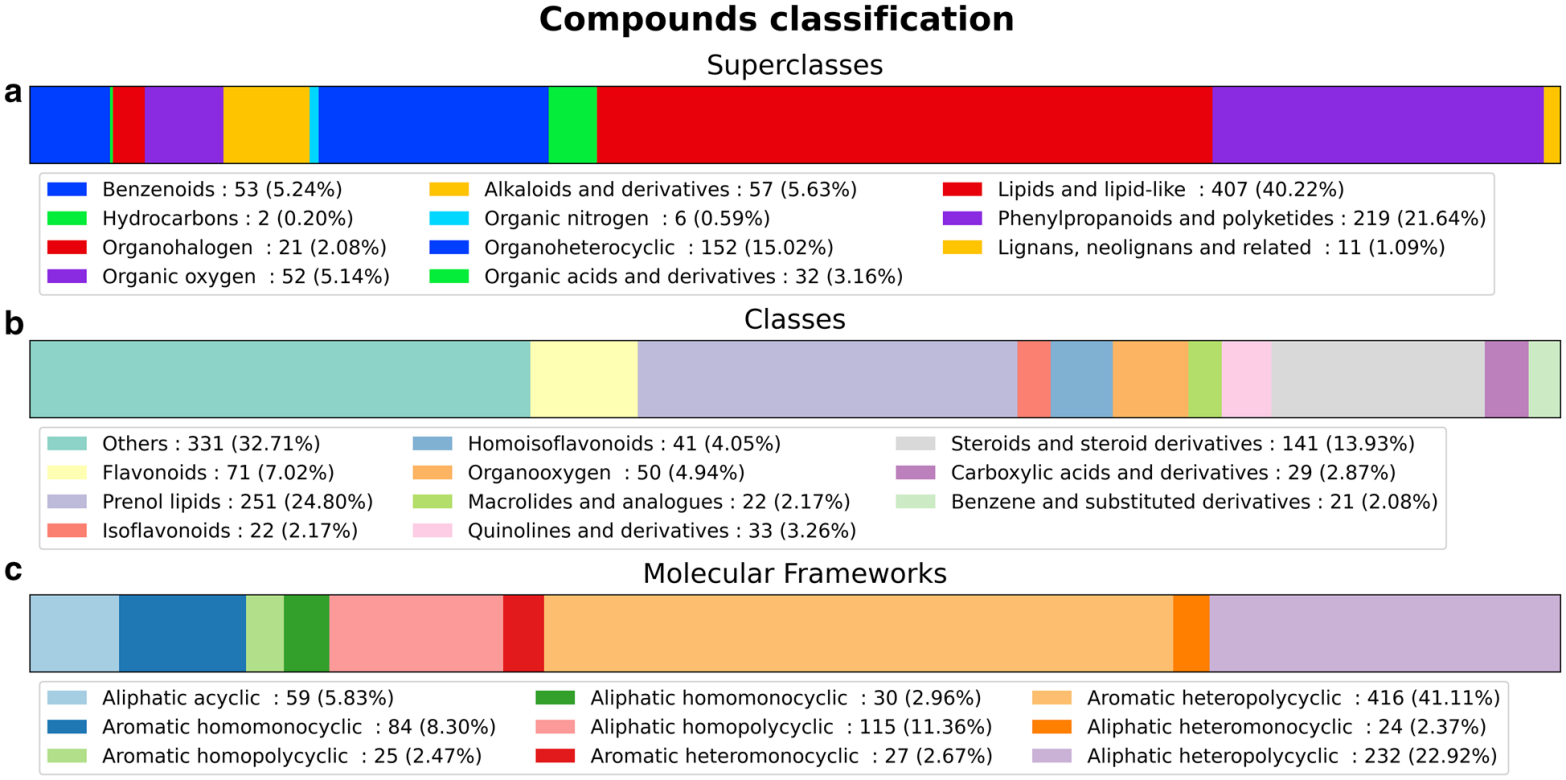
Stacked bar charts of the compound classifications. **a** SANCDB compounds superclasses. **b** SANCDB compounds classes. **c** SANCDB molecular frameworks. Classifications were obtained from ClassyFire

NPs classification is useful to assess their diversity, and can be done via different schemes [48, 78]. Here, ClassyFire, which performs a hierarchical classification using structural patterns into kingdoms, superclasses, classes and subclasses, was utilized [20]. We noted 11 superclasses out of the 26 ClassyFire organic compound superclasses; 77 classes out of 764 ClassyFire classes [35]; and 124 subclasses. These numbers indicate database diversity, and therefore potentiality for a variety of biological activities. The distribution of compounds superclasses is shown in Fig. 3a, classes in Fig. 3b and molecular frameworks in Fig. 3c. Top compound classes were the phenol lipids (251–24.8%), the steroids and steroid derivatives (141–13.9%), the flavonoids (71–7%), the organooxygen compounds (50–4.9%) and the homoisoflavonoids (41–4.1%).

Comparatively, SANCDB has a similar distribution to other compound databases. The Integrated Ethiopian Traditional Herbal Medicine and Phytochemicals Database (ETM-DB) [79] compounds classification was also done using ClassyFire, and shares the same top three superclasses. ETM-DB with 3930 compounds has 22 superclasses and 200 classes. The 500 Pan-African Natural Products Library (p-ANAPL) compounds are distributed across 30 classes [80] while the Nuclei of Bioassays, Ecophysiology and Biosynthesis of Natural Products Database (NuBBE) database has 14 classes with 2147 compounds [48]. NuBBE and p-ANAPL use however a different classification scheme. SANCDB contains 13.93% of steroids and derivatives, and was previously shown to have the highest rates of steroids compared to other NPs databases [81].

The database was rich in polycyclic compounds in Fig. 3c. Indeed, the molecular framework distribution showed that only 59 (5.8%) of the compounds were acyclic. Nine distinct types of molecular frameworks were found in SANCDB. The most common frameworks were the aromatic heteropolycyclic (416–41.1%), the aliphatic heteropolycyclic (232–22.9%) and the aliphatic homopolycyclic (115–11.4%). Molecular framework is similar to the concept of scaffold [44] and describes compounds according to their aliphaticity/aromaticity, ring count, and the diversity of atom types [35]. It is an important feature to assess compounds libraries' diversity in screening campaigns [26].

### Compounds activities

**Fig. 4:**
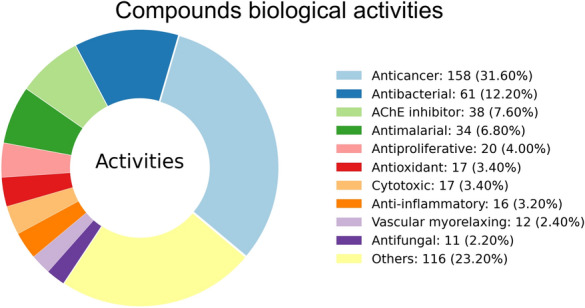
SANCDB compounds biological activities. A donut chart showing the distribution of various biological activities of a total of 318 compounds which have documented use in their references in SANCDB. The top 10 activities classes are shown while the remaining ones are grouped in the others category. The biological activities of the remaining compounds in the database is yet to be reported

From the source reference, where significant biological activity of isolated compounds was determined, the information was recorded and standardized to avoid duplication. The distribution of biological activities for the 318 compounds showed anticancer (158–31.6%), antibacterial (61–12.2%), AChE inhibitor (38–7.6%), antimalarial, (34–6.8%) and antiproliferative (20–4.0%) as most common activities (Fig. 4). We noted 59 distinct activity types. Some compounds showed a variety of activities (> 6 different activities): Combretastatin A-1, Ouabain, Acovenoside A, Isoorientin and Quercetin. They were also found to be associated with at least 15 predicted targets in ChEMBL [31] with 90% confidence. They can be a good starting point for multi-target inhibitors. It is noteworthy that activities assignment was not standardized. Recorded activities could be at the molecular, cellular, tissue or disease level. Also, recording of activities was limited to only the reference in the database. Furthermore, only compounds showing a significant level of activity were recorded, limiting the number of assigned biological activities to 318 out of the 1012 in the database.

### Commercially available analogs

A first evaluation of SANCDB NPs availability showed that only about 30% of SANCDB was readily purchasable. 316 were obtainable on MolPort [38] while 327 on Mcule [37]. A previous assessment of NPs commercial availability (in the ZINC subset of readily purchasable compounds) showed that only 10% were purchasable [3]. In general, NPs are insufficiently covered in commercial databases [3]. Additionally, 118 SANCDB compounds had synthetic accessibility [82] score greater than six (see Additional file 1: Fig. S1). They may thus pose synthetic challenges [82].

**Fig. 5:**
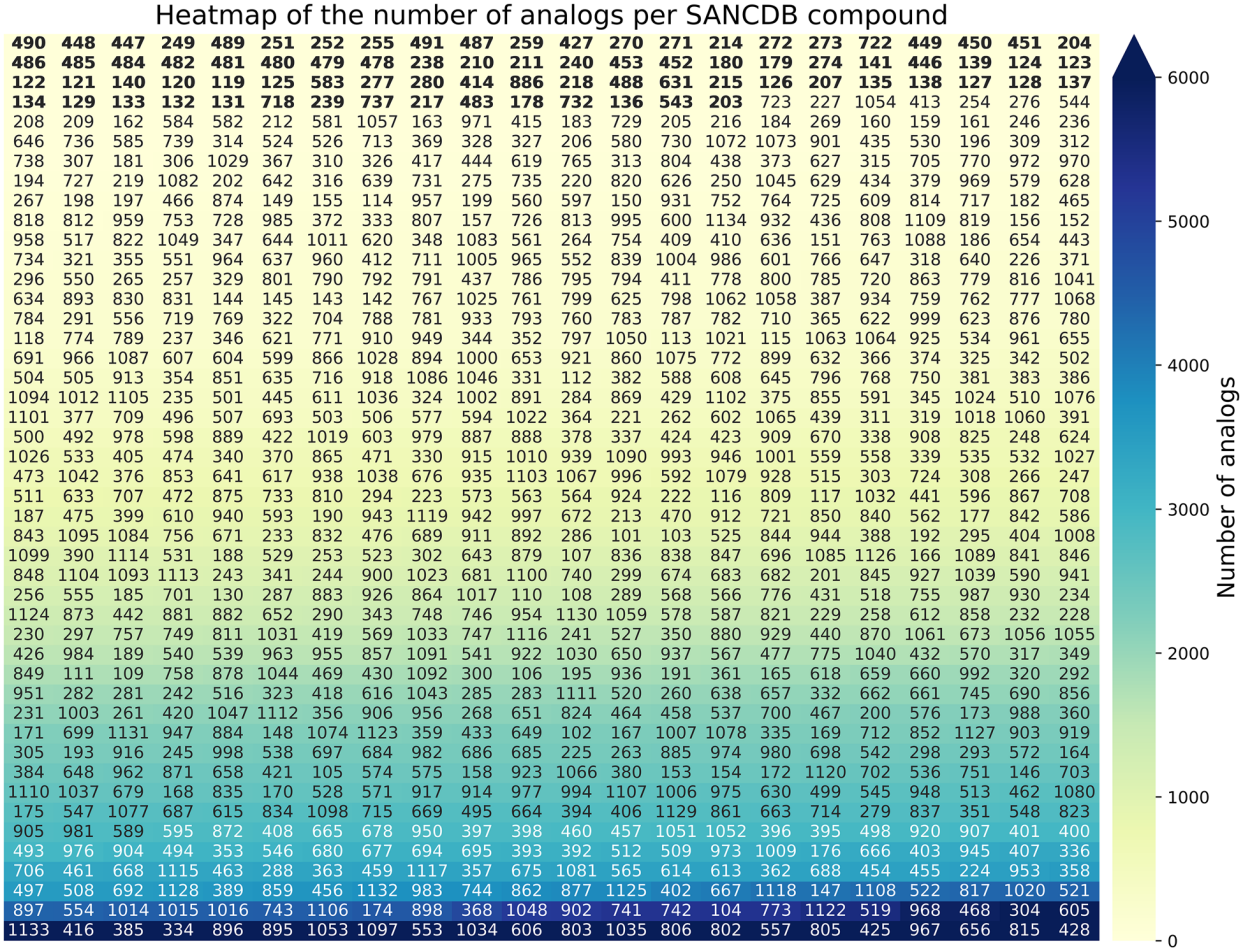
Number of analogs per SANCDB compound. Compound are ordered by their number of analogs (lowest to highest, left to right). Labels on the cells are the compounds IDs. For visibility purposes, the heatmap vmax is set to 6000. Yet some compounds have more than 6000 analogs (indicated by the arrow on the color key). IDs without analogs are in bold. Text color (compounds IDs) varies from white to black for readability. The color gradient represents the count of analogs

In the updated version of SANCDB, a total of 380,206 unique commercially available analogs were added with an average of 1487 analogs per compound (at the time of writing). 232,747 analogs were retrieved from Mcule and 141,320 from Molport. Each compound analogs’ SMILES and Tanimoto similarity scores are available for download. The downloaded Molport [38] and Mcule [37] databases contained 7,597,214 and 9,884,200 compounds, respectively in October 2019. The distribution of the number of analogs per compound is shown in Fig. 5. Frequencies ranged from 42,224 to zero analogs. SANC00428, SANC00815, SANC00656, SANC00967 and SANC00425 have the highest number of analogs with 42,224, 29,045, 27,823, 26,638, 24,993 analogs respectively. These compounds have low molecular weight (MW). All compounds with more than 10,000 analogs had a MW below 300 Da. However, there was no correlation between the number of analogs and the MW with a negligible Pearson correlation of -0.09 (see Additional file 1: Figs. S2 and S3).

No analog was found for 70 compounds using a Tanimoto similarity coefficient threshold as low as 0.6. SciFinder [27] database was used to manually retrieve analogs for these compounds. As SciFinder [27] search was per similarity interval (e.g. 0.75–0.79 similarity interval), only the first interval having analogs, starting from the highest, was considered. The number of analogs for these compounds ranged from one to 29 with 23 compounds having only one analog [27]. The number of analogs per compounds and their respective similarity thresholds are available in Additional file 1: Table S1. Also, only 43% (442) of the compounds had more than 1000 analogs in Molport [38] and Mcule [37] datasets. This indicates a low coverage of NPs availability considering the size of the datasets used (Molport and Mcule with 7,597,214 and 9,884,200 respectively) and the low Tanimoto similarity coefficient cutoff used (0.6).

**Fig. 6:**
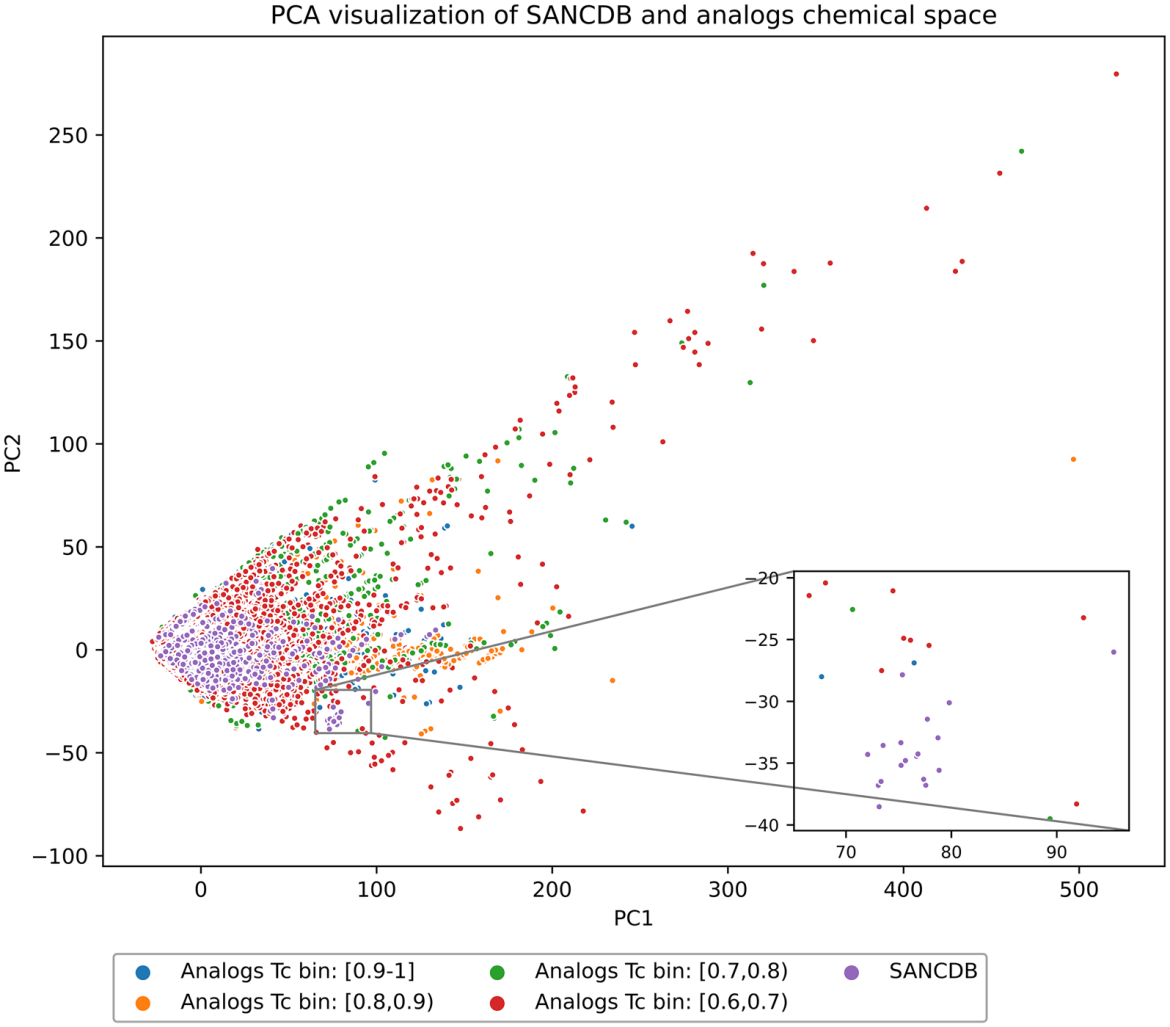
PCA visualization of SANCDB and analogs chemical space. Compounds (n = 375,061) are represented in dots. SANCDB (violet, n = 1012). Analogs are in bins of similarity values: (0.6,0.7) blue, n = 266,147; (0.7,0.8) orange, n = 69,336; (0.8,0.9) green, n = 24,679; [0.9,1] red, n = 13,887. As an analog may have different similarity scores with different SANCDB compounds, the maximum similarity score was chosen for each analog. The first two components explain 81% of the variance (PC1 (66%), PC1 (15%))

Analogs covered SANCDB chemical space regions (Fig. 6). SANCDB compounds formed a cluster overlapped with analogs which extend by decreasing similarity score. Analogs with similarity values in the range (0.6, 0.7) were the most isolated. Some SANCDB compounds without analogs occupied a small isolated cluster (zoomed region of the plot). Further analysis showed that they corresponded to compounds with zero analogs. The t-SNE visualization showed a less dense cloud, thus indicating further separation between compounds (see Additional file 1: Fig. S4).

SANCDB analogs can expand initial drug discovery projects. Analog information is important during hit optimization in drug discovery process. Screening hits identified from SANCDB can be further optimized through their analogs [13, 16, 17, 19, 83]. Additionally, more potent analogs of the potential allosteric modulators identified in SANCDB [14, 15, 84] may further enhance allosteric modulation of these compounds on their targets.

### Scaffolds and compounds subsets

SANCDB compounds were analyzed in terms of their scaffolds and with regard to different subsets of chemical compounds relevant to drug discovery. NPs scaffolds are of interest as they are rich in sp^3^-configured centers while synthetic scaffolds are generally flatter [80, 85]. They also often serve as the basis for synthetic modifications of drug-like compounds [65]. Scaffold diversity is ideal for screening libraries as virtual screening also aims to find new scaffolds [66].

**Fig. 7:**
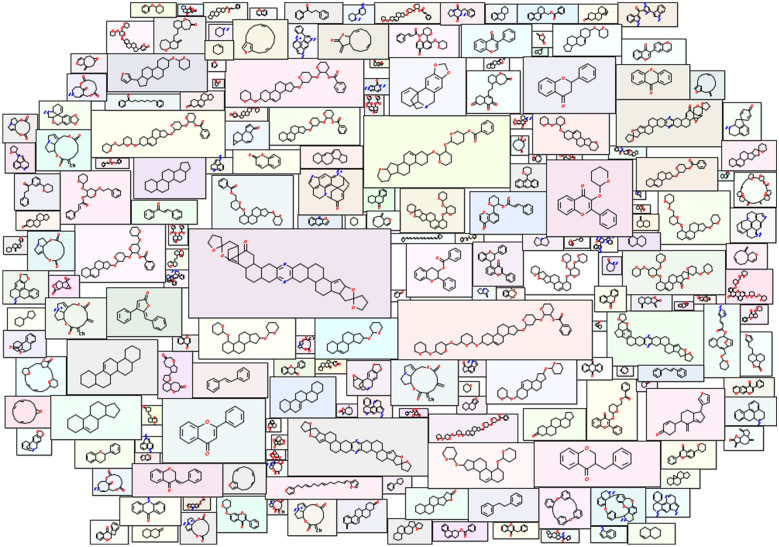
Molecule cloud of SANCDB scaffolds. Structure sizes indicate scaffold frequencies. The benzene ring is a special case, being the most frequent scaffold in all large data sets [48]. Therefore, it is not displayed. A figure with the most common scaffolds and their frequencies is presented in Fig. S5

The molecule cloud visualization in Fig. 7 highlights top-ranked scaffolds. It also helps assess the diversity of scaffolds and their structural features, allowing the reader a rapid overview of the most common scaffolds [47]. However, a drawback may be the less visible of the less common scaffolds which may still be of interest. All scaffolds and their count are presented in Additional file 1: Table S2.

In SANCDB, about half of the compounds presented a unique scaffold. Indeed, 501 unique scaffolds were identified from the 1012 compounds, indicating their diversity. 59 compounds did not present a scaffold as they were purely aliphatic. Scaffold frequencies followed a “long tail” distribution (see Additional file 1: Fig. S5) common in compound datasets [47]. This shows the high number of singletons, compounds containing a unique scaffold in the entire database, highlighting this latter diversity.

Most common scaffolds were flavonoids, already known to be common in NP datasets [44]. Interestingly, they were only the third most represented in the distribution of compound classes in Fig. 3. This may be related to a structural diversity in the first two categories compared to flavonoid which may be more homogeneous. Structures of the top 10 scaffolds are represented in Additional file 1: Fig. S6. The chromane 3-Benzylchroman-4-one was the most common scaffold with 30 compounds. Its structure presents a bicycle consisting of a 3,4-dihydro-1-benzopyran, with a ketone group which is a structural alert. Structural alerts are high reactive groups which may cause toxicity [86]. The compound is known for human monoamine oxidase B inhibition [87]. Chromane scaffolds are promiscuous in NPs [67] and known for their anticancer activity [88] which may also be related with the database richness in anticancer compounds in Fig. 4. The second most abundant was flavone found in 21 compounds. Its prodrug aminoflavone reached phase 2 clinical trials for breast cancer treatment [87]. The related compound in the database may present similar activity. Finally, flavanone was the third most common scaffold with 14 compounds. This scaffold also presented a ketone group as a structural alert.

**Fig. 8:**
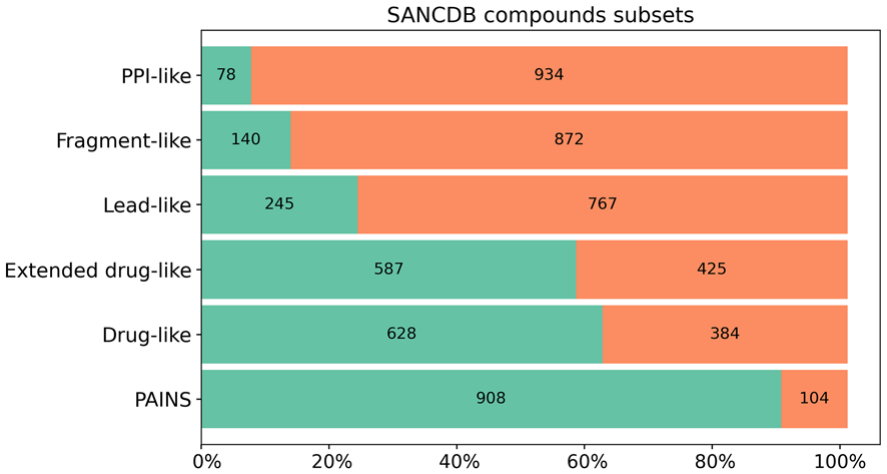
Proportions of compounds in each subset (drug-like, extended drug-like, fragment-like, lead-like, PPI-like). The count of each subset and their related proportions on the x-axis are reported. The green area indicates compounds fitting into that category. In the PAINS group, it indicates compound free of PAINS patterns

Over half of the database was drug-like or extended drug-like compounds, shown in Fig. 8. We noted a minor difference (41 compounds) between the drug-like and extended drug-like compounds, with the latter having only two more conditions to the drug-like category. PPI-like and fragments-like subsets represent the most stringent conditions for the database compounds, hence showing the least number of compounds. NP datasets may have a low proportion of fragments due to their polycyclic nature. As shown by the compound classification, SANCDB was rich in polycyclic compounds (~ 75% of the compounds see Fig. 3c). Thus, a low proportion of fragments is expected. PPI-like compounds (MW > 400 and logP > 4) represented the smallest set with only 78 compounds. This contrasts with the high proportion of polycyclic compounds in the database. Given these low proportions of fragment-like and PPI-like compounds, the database may not be suitable for fragment-based drug discovery or protein–protein inhibition.

These proportions were similar to those observed in many other NPs databases with drug-like and extended drug-like being more than 50% while PPI-like, fragments-like and PAINS have low proportions [45].

These subsets fit different contexts in drug discovery. For example, fragments can easily be found in early stage screening to identify potent chemotypes for latter optimization. PPI-like are ideal candidates to block protein–protein interaction. The distribution of the different subsets can also be a good indicator of the ideal context for a dataset. For example, for a database enriched in fragments, fragment-based drug discovery approaches might be ideal. Small molecule databases for screening such as ZINC [33] are often subdivided into subsets. PAINS patterns are used to filter out frequent hitters in screening [89]. Hence, the various SANCDB subsets can be used to establish custom-made virtual screening experiments based on user’s specific demands.

## Conclusions

NPs remain an integral component of the drug discovery process. Hitherto, a large proportion of approved drugs have been derived from NPs [5]. This has inspired the establishment of numerous databases which contain diverse chemical classes of NPs to facilitate bioprospecting of important leads for biomedical and chemical research [1, 3]. Since the establishment of SANCDB in 2015, its usage as a source of data for both in silico screening and machine learning has been on the rise. Thus, to maintain its relevance, the current work aimed to update the database with additional compounds isolated from South African natural resources, as well as to add new functionalities aimed at providing a larger chemical space for hit exploration. To this end, the updated fully referenced relational database contains more than 1000 unique compounds from South Africa. A classification and scaffold analysis showed a diverse chemical representation with 501 unique NP scaffolds. The chemical diversity of a database is an indicator of how useful it can be for hit identification [25]. The database dataset is freely accessible and is downloadable in different chemical formats including ready to dock ones using either AutoDock [58] or Maestro from Schrödinger [37]. In consideration of the universally acknowledged limitations of NPs as a result of their complex structural organization, the current update also includes the incorporation of readily available analogs from two main commercial chemical databases (MolPort [38] and Mcule [37]). In comparison to other existing NP databases, this feature is present only in SANCDB and will provide users with a larger chemical library of compounds for both chemoinformatic and bioscreening studies. The analogs from the different databases are constantly updated via an automated pipeline making it more reliable. Analogs have been linked to their sources on Mcule [37] and MolPort [38], allowing users to obtain compounds for in vitro screening seamlessly.

## Supplementary Information

**Additional file 1:** **Fig S1.** Distribution of SAscore for SANCDB compounds. X-axis represents SAscores and y-axis quantifies the corresponding probability densities. **Fig. S2.** Scatter plot of compounds molecular weight (MW) versus analogs count. X-axis and y-axis correspond to MW (Dalton) and the number of analogs respectively. **Fig. S3.** Scatter plot of compounds molecular weight (MW) versus analogs count. **Fig. S4.** t-SNE visualization of SANCDB and analogs chemical space. **Fig. S5.** Histogram and kernel density distribution of the scaffolds count. **Fig. S6.** Top 10 SANCDB scaffolds structures and their counts. **Table S1.** SANCDB analogs from Sci-finder for compounds without analogs on Mcule and Molport chemical databases. **Table S2.** A summary of all scaffold structures in SANCDB database.

## Data Availability

All data generated or analysed during this study are included in this published article. Supplementary information accompanies this article. SANCDB is freely available at https://sancdb.rubi.ru.ac.za/.

## Abbreviations

API: Application Programming Interface
AChE: Acetylcholinesterase
CAS: Chemical Abstracts Service
DOI: Digital Object Identifier
ETM-DB: Integrated Ethiopian Traditional Herbal Medicine and Phytochemicals Database
GBIF: Global Biodiversity Information Facility
MMFF94: Merck Molecular Force Field
MQN: Molecular Quantum Numbers
MW: Molecular weight
NaPLeS: Natural products likeness scorer
NP: Natural Product
NPASS: Natural Product Activity and Species Source
nHA: Number of hydrogen bond acceptor
nHD: Number of hydrogen bond donor
nRing: Number of rings
nRot: Number of rotatable bonds
NuBBE: Nuclei of Bioassays, Ecophysiology and Biosynthesis of Natural Products Database
p-ANAPL: Pan-African Natural Products Library
PPI-like: Protein–protein inhibitor like
RUBi: Research Unit in Bioinformatics
SANBI: South African National Biodiversity Institute
SANCDB: South African natural compound database
TPSA: Total Polar Surface Area
t-SNE: T-Distributed Stochastic Neighbor Embedding
