# Supplementary material for: SANCDB: an update on South African natural compounds and their readily available analogs
Source: J Cheminform. 2021 May 5;13:37. doi: 10.1186/s13321-021-00514-2 (PMC8097257; doi:10.1186/s13321-021-00514-2)
Supplement: Supplementary file 1 — Additional file 1: Fig S1. Distribution of SAscore for SANCDB compounds. X-axis represents SAscores and y-axis quantifies the corresponding probability densities. Fig. S2. Scatter plot of compounds molecular weight (MW) versus analogs count. X-axis and y-axis correspond to MW (Dalton) and the number of analogs respectively. Fig. S3. Scatter plot of compounds molecular weight (MW) versus analogs count. Fig. S4. t-SNE visualization of SANCDB and analogs chemical space. Fig. S5. Histogram and kernel density distribution of the scaffolds count. Fig. S6. Top 10 SANCDB scaffolds structures and their counts. Table S1. SANCDB analogs from Sci-finder for compounds without analogs on Mcule and Molport chemical databases. Table S2. A summary of all scaffold structures in SANCDB database. [file 13321_2021_514_MOESM1_ESM.docx]

SANCDB: an update on South African natural compounds and their readily available analogs

Bakary N’tji Diallo^1^, Michael Glenister^1^, Thommas M. Musyoka^1^, Kevin Lobb^1,2^, and Özlem Tastan Bishop^1,*^

^1^ Research Unit in Bioinformatics (RUBi), Department of Biochemistry and Microbiology, Rhodes University, Makhanda/Grahamstown, 6140, South Africa

^2^ Department of Chemistry, Rhodes University, Makhanda/Grahamstown, 6140, South Africa

***** Correspondence: o.tastanbishop@ru.ac.za; Tel.: +27-46-603-7576 (O. T. B)

*Supplementary Material*


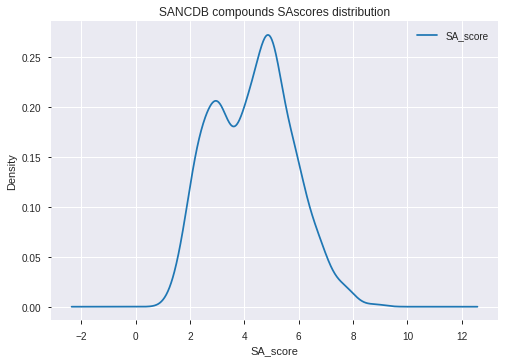


**Fig S1:** Distribution of SAscore for SANCDB compounds. X-axis represents SAscores and y-axis quantifies the corresponding probability densities.


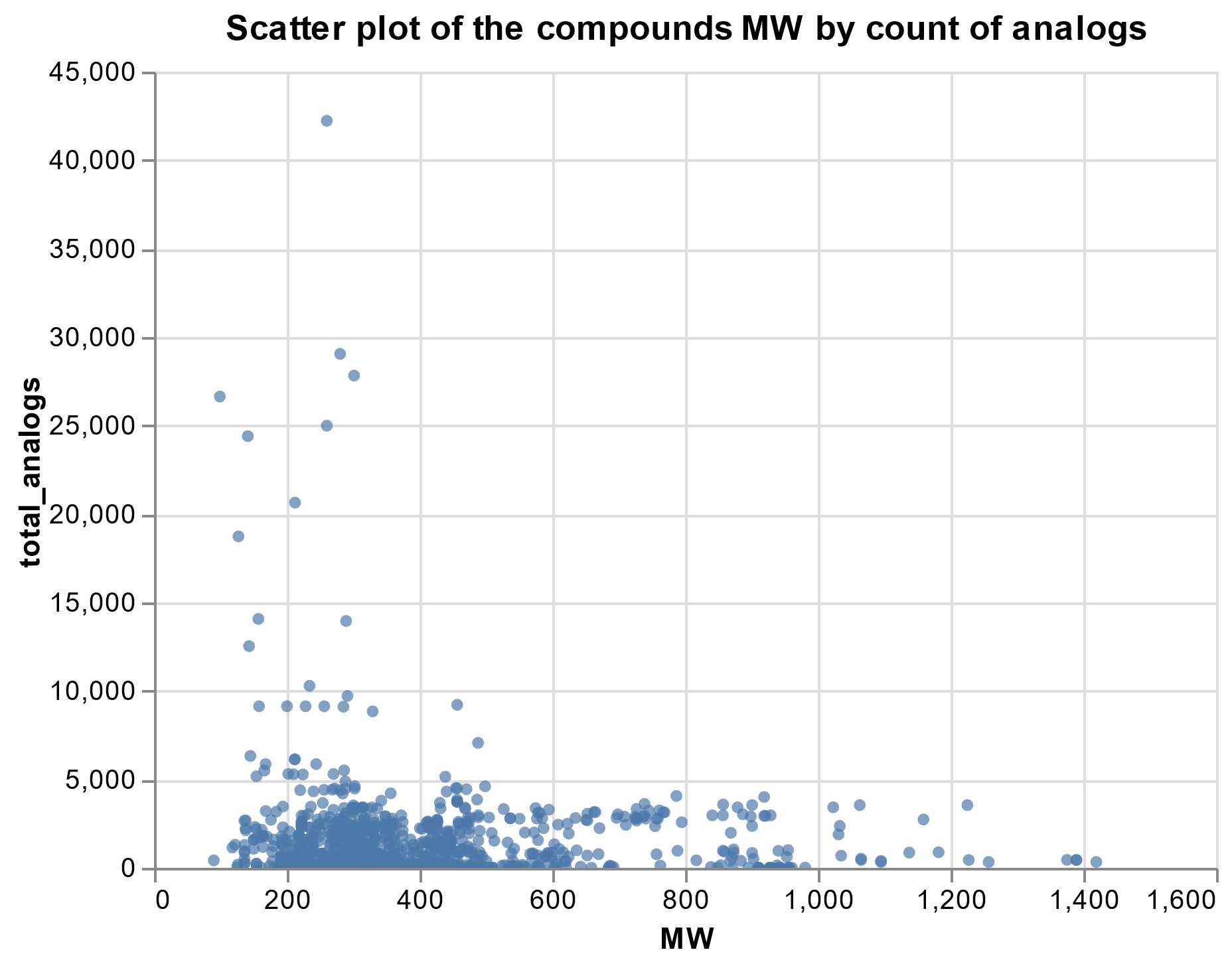


**Fig. S2:** Scatter plot of compounds molecular weight (MW) versus analogs count. X-axis and y-axis correspond to MW (Dalton) and the number of analogs respectively.


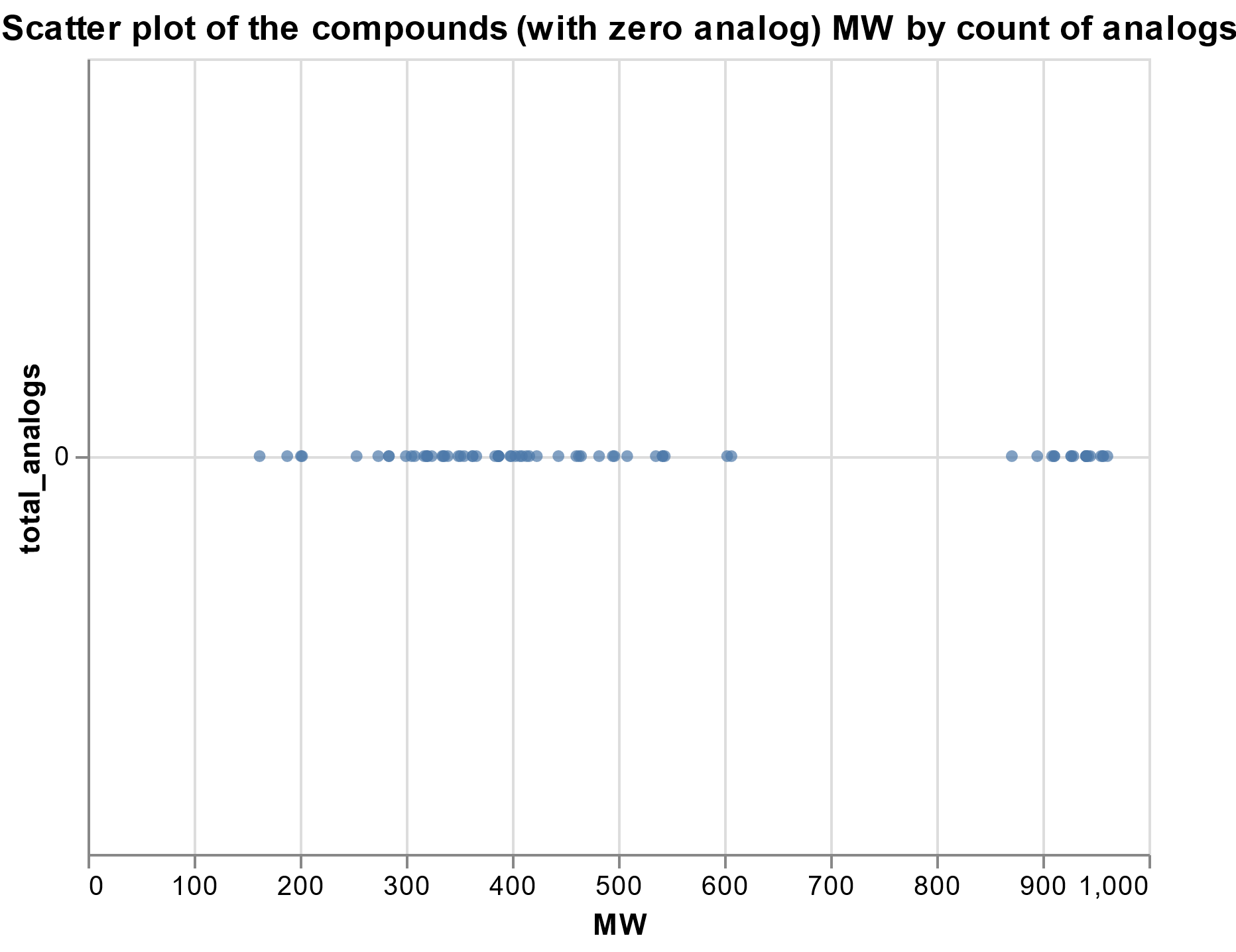


**Fig. S3:** Scatter plot of compounds molecular weight (MW) versus analogs count. X-axis and y-axis correspond to MW (Dalton) and the number of analogs respectively. Only compounds with zero analog count are shown.


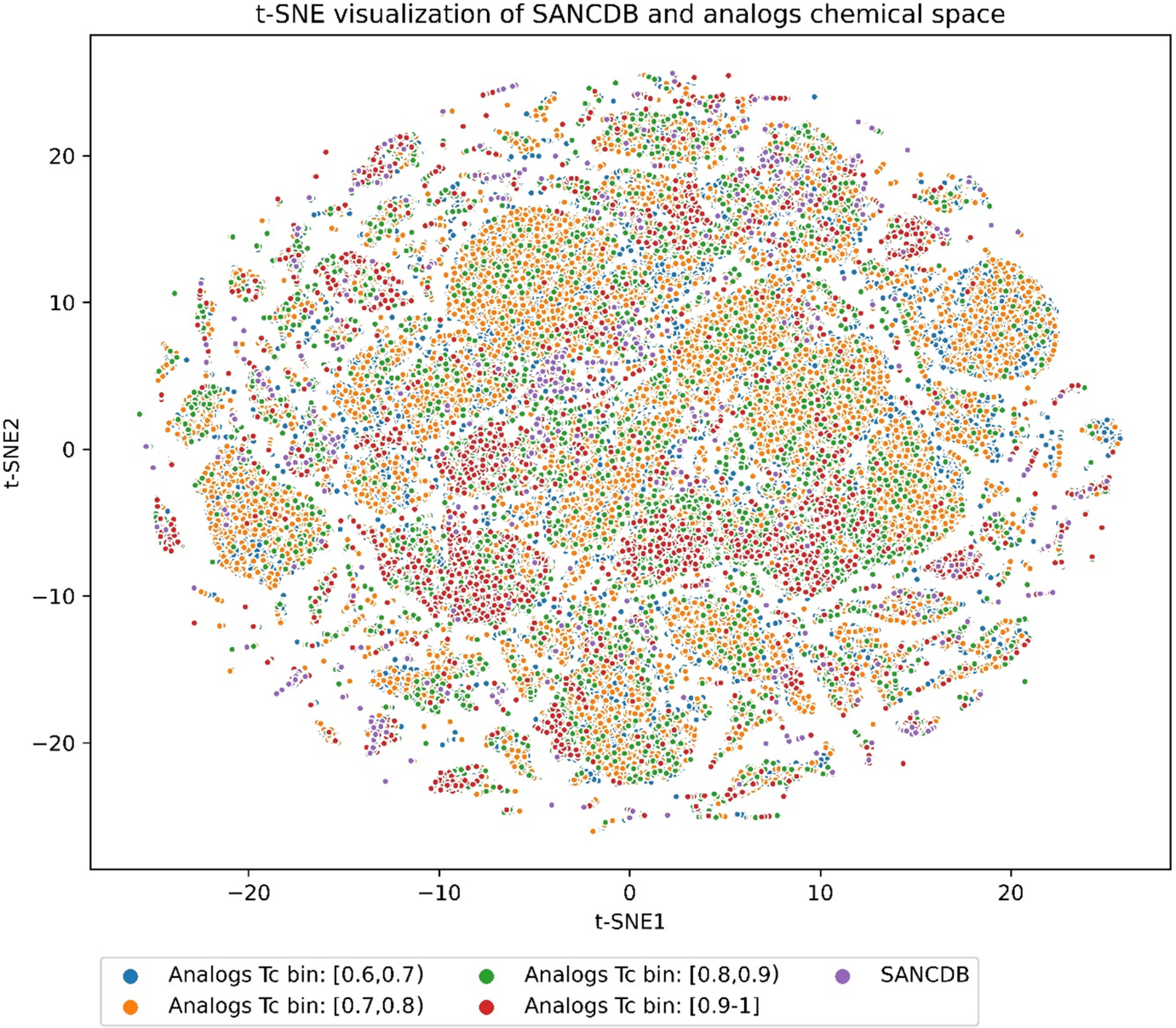


**Fig. S4:** t-SNE visualization of SANCDB and analogs chemical space. Compounds (n = 375061) are represented in dots. SANCDB (violet, n = 1012). Analogs are in bins of similarity values [0.6,0.7) (blue, n = 266147), [0.7,0.8) (orange, n = 69336), [0.8,0.9) (green, n =24679), [0.9-1] (red, n = 13887). As an analog may have different similarity scores with different SANCDB compounds, the maximum similarity score was chosen for each analog.


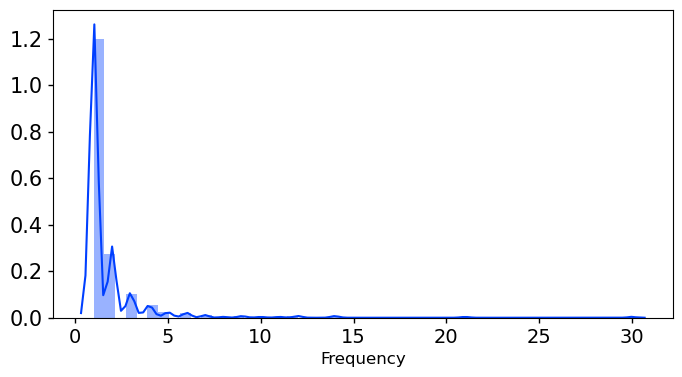


**Fig. S5:** Histogram and kernel density distribution of the scaffolds count.


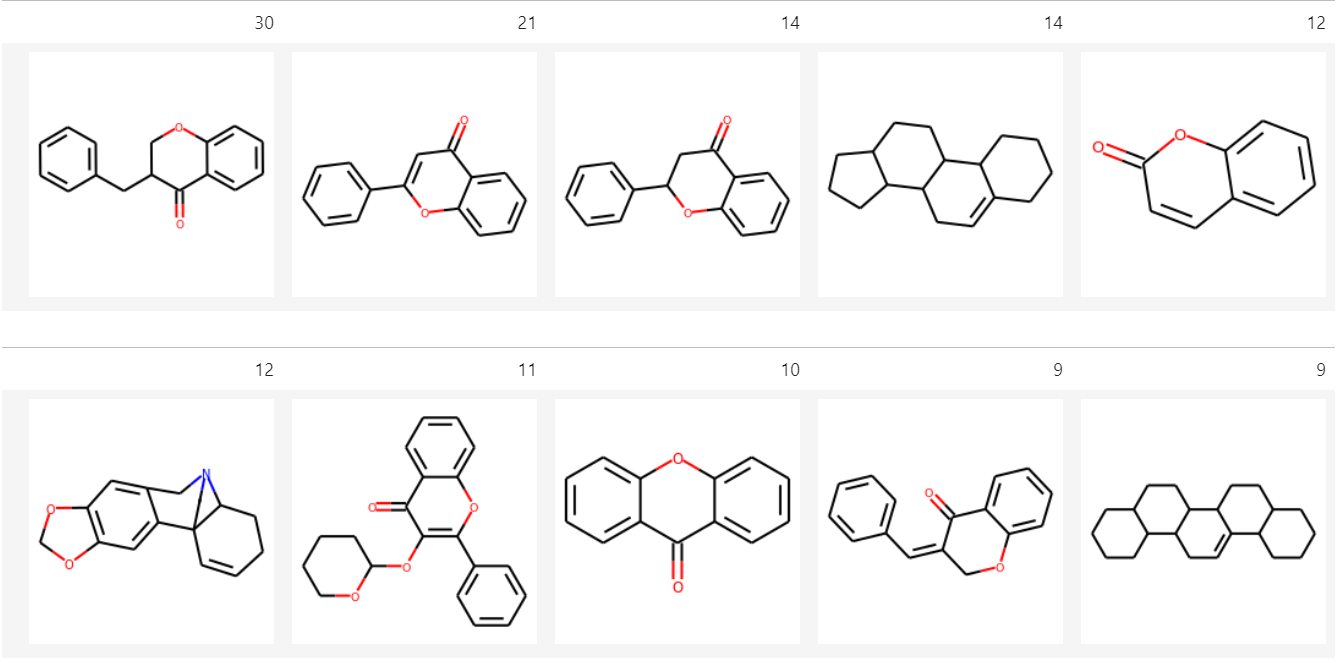


**Fig. S6:** Top 10 SANCDB scaffolds structures and their counts.

**Table S1.** SANCDB analogs from Sci-finder for compounds without analogs on Mcule and Molport chemical databases. *n* represents the number whereas ST is similarity threshold used.

| SANCDB_ID | *n* | ST | SANCDB_ID | *n* | ST |
| --- | --- | --- | --- | --- | --- |
| SANC00204 | 1 | 85-89 | SANC00138 | 3 | 85-89 |
| SANC00123 | 1 | 90-94 | SANC00180 | 1 | 99-100 |
| SANC00272 | 6 | 85-89 | SANC00120 | 1 | 90-94 |
| SANC00124 | 4 | 80-84 | SANC00240 | 2 | 85-89 |
| SANC00480 | 15 | 70-99 | SANC00121 | 1 | 99-100 |
| SANC00486 | 24 | 80-99 | SANC00215 | 4 | 99-100 |
| SANC00127 | 1 | 90-94 | SANC00139 | 1 | 90-94 |
| SANC00238 | 1 | 99-100 | SANC00478 | 16 | 90-99 |
| SANC00453 | 9 | 70-99 | SANC00446 | 25 | 70-99 |
| SANC00255 | 1 | 99-100 | SANC00249 | 1 | 99 |
| SANC00451 | 16 | 70-99 | SANC00448 | 24 | 70-99 |
| SANC00484 | 20 | 80-99 | SANC00270 | 2 | 90-94 |
| SANC00207 | 4 | 95-98 | SANC00485 | 18 | 80-99 |
| SANC00427 | 11 | 70-99 | SANC00273 | 13 | 80-99 |
| SANC00140 | 3 | 99-100 | SANC00125 | 1 | 75-79 |
| SANC00482 | 17 | 90-99 | SANC00135 | 2 | 70-74 |
| SANC00251 | 1 | 85-89 | SANC00214 | 2 | 99-100 |
| SANC00134 | 1 | 85-89 | SANC00490 | 25 | 90-99 |
| SANC00483 | 19 | 80-99 | SANC00218 | 3 | 85-98 |
| SANC00122 | 1 | 99-100 | SANC00141 | 1 | 95-98 |
| SANC00450 | 24 | 70-99 | SANC00487 | 17 | 80-99 |
| SANC00447 | 25 | 70-99 | SANC00271 | 2 | 90-94 |
| SANC00479 | 15 | 90-99 | SANC00722 | 12 | 80-99 |
| SANC00252 | 1 | 90-94 | SANC00481 | 23 | 90-99 |
| SANC00119 | 1 | 90-94 | SANC00131 | 1 | 99-100 |
| SANC00132 | 1 | 75-79 | SANC00128 | 4 | 85-89 |
| SANC00489 | 25 | 85-99 | SANC00274 | 16 | 80-99 |
| SANC00452 | 29 | 65-99 | SANC00133 | 3 | 85-89 |
| SANC00210 | 4 | 95-98 | SANC00239 | 1 | 99-100 |
| SANC00737 | 5 | 80-99 | SANC00631 | 13 | 85-99 |
| SANC00488 | 16 | 90-99 | SANC00129 | 2 | 99-100 |
| SANC00126 | 1 | 75-79 | SANC00280 | 12 | 75-99 |
| SANC00414 | 15 | 75-99 | SANC00211 | 1 | 80-84 |
| SANC00179 | 2 | 85-89 | SANC00583 | 10 | 90-99 |
| SANC00178 | 1 | 99-100 | SANC00137 | 2 | 90-94 |

**Table S2**. A summary of all scaffold structures in SANCDB database. The value alongside (n) represents the total number of compounds containing the indicated scaffold.

| Scaffold | *(n)* | Scaffold | *(n)* |
| --- | --- | --- | --- |
| c1ccccc1 | 51 | C1=Nc2c3c(c[nH]c3cc3[nH]cc(-c4ccccc4)c23)C1 | 1 |
| O=C1c2ccccc2OCC1Cc1ccccc1 | 30 | C1=C2CCCCC2C2CCC3CC(OC4CC(OC5CCCCO5)CCO4)CC3C2C1 | 1 |
| O=c1cc(-c2ccccc2)oc2ccccc12 | 21 | O=C(OC1CC2COC3(CCC(=O)C(OC(=O)c4ccoc4)C13)C2)c1ccoc1 | 1 |
| O=C1CC(c2ccccc2)Oc2ccccc21 | 14 | O=C1C=CC2(CC=Nc3cc4[nH]cc5c4c(c32)N=CC5)CC1 | 1 |
| C1=C2CCCCC2C2CCC3CCCC3C2C1 | 14 | O=C(C=Cc1c[nH]cn1)OC1CC2C=CCCC2C=CC2C=CC1O2 | 1 |
| O=c1ccc2ccccc2o1 | 12 | C1=CC2CCCC2C(OC2CCCCO2)O1 | 1 |
| C1=CC23CCN(Cc4cc5c(cc42)OCO5)C3CC1 | 12 | O=c1ccc(C2CCC3C2CCC2C4CCC=CC4=CCC23)co1 | 1 |
| O=c1c(OC2CCCCO2)c(-c2ccccc2)oc2ccccc12 | 11 | O=C1CCCO1 | 1 |
| O=c1c2ccccc2oc2ccccc12 | 10 | C1=C2C3CN(Cc4cc5c(cc43)OCO5)C2CCC1 | 1 |
| O=C1C(=Cc2ccccc2)COc2ccccc21 | 9 | c1cc2cc3cc4c(cc3nc2o1)OCO4 | 1 |
| C1=C2C3CCCCC3CCC2C2CCC3CCCCC3C2C1 | 9 | C1=C2CCCCC2C2CCC3CCCC3C2=C1 | 1 |
| O=C1CC2C(CCC3Cc4nc5c(nc4CC32)CC2CCC3C4=CC6OC7(CCCO7)CC6C4CCC3C2C5)C2=CCC3CC4(CCCO4)OCC123 | 8 | C1=C2CC(OC3CCC(OC4CCC(OC5CCC(OC6CCCCO6)CO5)CO4)CO3)CCC2C2CCC3CCCC3C2C1 | 1 |
| C1=CC2CCCCC2CC1 | 7 | O=C1CC2OCC3CC(=O)C4C(CCC5C(c6ccoc6)OC(=O)C6OC654)C32CO1 | 1 |
| C(=Cc1ccccc1)c1ccccc1 | 7 | C1=C2CCN3Cc4cc5c(cc4C(CC1)C23)OCO5 | 1 |
| C=C1C(=O)OC2C=CCCC=CCCC12 | 7 | O=c1c(-c2ccc3c(c2)C=CCO3)coc2cc3c(cc12)CCCO3 | 1 |
| C1=CCCCC1 | 6 | c1ccc(Cc2occ3ccccc23)cc1 | 1 |
| c1ccc(CCc2ccccc2)cc1 | 6 | O=C1C=C2CCCC2C2CC2C1 | 1 |
| C1CC2CCCN2C1 | 6 | O=c1cc(-c2ccccc2)oc2c(C3CCCCO3)cccc12 | 1 |
| O=c1c2ccccc2[nH]c2ccccc12 | 6 | C1CCC(OC2CCCOC2OC2CCCOC2OC2CCC3C(CCC4C3CCC3C5CCCOC5CC34)C2)OC1 | 1 |
| O=c1c(-c2ccccc2)coc2ccccc12 | 6 | O=C1C=CC2(c3ccccc3)CCNC2C1 | 1 |
| O=C1C=C(c2ccccc2)C(=Cc2ccccc2)O1 | 6 | C=C1CCCCC12CCCCC2 | 1 |
| C1CCC2C(C1)CCC1C2CCC2C3CCCC3CCC21 | 5 | C=C1C(=O)OC2C=CCCCC=CCC12 | 1 |
| [CH]=C1CC(=C)CC(=O)OCC2=CCN3CCC(OC1=O)C23 | 5 | O=C1C=CC(=O)c2ccccc21 | 1 |
| C=C1C2=CCC(c3ccoc3)C2CCC1C1C=CC(=O)CC1 | 5 | O=C1Oc2ccccc2C(c2ccccc2)C1C1C(=O)Oc2ccccc2C1c1ccccc1 | 1 |
| O=C1C=C2SC3CC24CC1N1CCC2=c5c(cc(c4c51)=[NH+]3)N=C2 | 5 | O=c1cccc2n1CC1CC2CN2CCCCC12 | 1 |
| C1=CCC2CCCCC2C1 | 5 | O=C1C(=O)c2ccccc2C2=C1CCO2 | 1 |
| C1=C2CCCC(OC3CCCCO3)C2C2CCC3CC(OC4CCCCO4)CC3C2C1 | 5 | c1cc2cc(c1)Oc1ccc(cc1)CCc1ccccc1-c1ccc(cc1)CC2 | 1 |
| C1=CCCCc2coc(c2)CC=CCCC1 | 5 | c1cc2c3c(c1)Oc1ccc(cc1)CC1NCCc4cccc(c41)Oc1ccc(cc1)CC3NCC2 | 1 |
| O=C(C=Cc1ccccc1)c1ccccc1 | 4 | C1=C2CCCCC2CCC1 | 1 |
| c1ccc2c(c1)CC1NCCc3cccc-2c31 | 4 | C=c1cccc2c1=CC=C1C2CCC2C1CCC1C(=O)CCCC12 | 1 |
| C=C1CCC2CCCCC2C1 | 4 | O=C1C=C(CCc2ccoc2)C2CCCCC2C1 | 1 |
| c1ccc(C2CCc3ccccc3O2)cc1 | 4 | O=c1c2ccccc2oc2c3c(ccc12)OCC=C3 | 1 |
| O=C(OC1CCC(OC2CCCOC2OC2CCC3C(=CCC4C3CCC3C5CCCOC5CC34)C2)OC1)c1ccccc1 | 4 | c1cc(-c2cn(CCCN3CCCCCCCCC3)cc2-c2cccc3ncccc23)c2cccnc2c1 | 1 |
| C1=C2CCCCC2C2CCC3CC(OC4CCCCO4)CC3C2C1 | 4 | O=C(NC1C2COC1C1OC13CC1=CCC4CCCC4C1CCC23)c1ccccc1 | 1 |
| C1CCNCC1 | 4 | O=c1c2cc3c(cc2c2cccc4ccn1c42)OCO3 | 1 |
| C(=Cc1ccccc1)Cc1ccccc1 | 4 | C1CCC2C(C1)CCC1C3CCCC3CCC21 | 1 |
| C1CCC(OC2CC3CCC4C(CCC5CCCC(OC6CCCCO6)C54)C3C2)OC1 | 4 | C=C1CCCC2CC=CCC12 | 1 |
| [CH]=C1CC(=C)CC(=O)OCC2=CC[NH+]3CCC(OC1=O)C23 | 4 | c1ccc(Cc2ccccc2)cc1 | 1 |
| O=C(OC1Cc2ccccc2OC1c1ccccc1)c1ccccc1 | 4 | C1=NCCCC1 | 1 |
| [CH]=C1CCCC(=O)OCC2=CCN3CCC(OC1=O)C23 | 4 | O=C(C=Cc1ccccc1)OCC1CCCC(c2cccc3c(=O)ccoc23)O1 | 1 |
| C1=C2CCCCC2CCC2C1=CCC1CCCC12 | 4 | O=c1cncc(Cc2c[nH]c3ccccc23)[nH]1 | 1 |
| c1ccc2[nH]ccc2c1 | 4 | C=C1CCC2CC2C2CCCC12 | 1 |
| c1cc(C2CCC3C2CCC2C4CCC(OC5CCCCO5)CC4CCC23)co1 | 4 | C1=C2CC(OC3CCCCO3)CCC2C2CCC3CC(OC4CC(OC5CCCCO5)CCO4)CC3C2C1 | 1 |
| C1=C2C(CCC3C2CCC2CCCCC23)C2CCCCC2C1 | 4 | O=C1CC=CC2CC(OC3CCCCO3)CC(CCC=CC=CCC3CCC(CCCO1)O3)O2 | 1 |
| C1CCOCC1 | 3 | c1ccc2c(c1)CC(c1ccc3c(c1)OCO3)O2 | 1 |
| C1=C2C(CCC3C2CCC2Cc4nc5c(nc4CC23)CC2CCC3C4=CC6OC7(CCCO7)CC6C4CCC3C2C5)C2CC3(CCCO3)OC12 | 3 | C=C1C(C2CCOC(=O)CC2)CCC2C1C(=O)CC2c1ccoc1 | 1 |
| [CH]=C1CCCC(=O)OCC2=CC[NH+]3CCC(OC1=O)C23 | 3 | C1=C2CCCCC2c2ccccc2C1 | 1 |
| c1ccc2c(c1)CC1NCCc3cc4c(c-2c31)OCO4 | 3 | C1=C2C(CCC34CCCC(C3)O4)CCCC2OCCC1 | 1 |
| C1=CCCC=CCc2cc(co2)CCC=C1 | 3 | C1=C2C3CCCCC3CCC2C2CCCCC2C1 | 1 |
| O=C1CCCCC(=O)OC2CCN3CC=C(CO1)C23 | 3 | O=C1C=C2CCC34C=CC(CCC3C2CC1=O)C4 | 1 |
| C1=CCCC=CCCC2OC2CCC=C1 | 3 | O=C1OC2CCCOC2c2ccccc21 | 1 |
| c1ccc2nc3occc3cc2c1 | 3 | O=C1OC(C=Cc2ccccc2)Cc2ccccc21 | 1 |
| O=C1CCC2C(CCC3C2CCC2C4CCCC4CCC23)C1 | 3 | O=C1CCC23C(=O)CCC4CC45OC(C=C12)CC35 | 1 |
| O=C1OC2Cc3ccccc3C13CCCCC23 | 3 | O=C1CCC=CCCCOC(=O)CNC(=O)C(Cc2ccccc2)NC(=O)CN1 | 1 |
| c1c2c(cc3c1OCO3)C13CCN(C2)C1CCC1OC13 | 3 | O=C1C=C2SC3CC24c2c5c6c(cc2=[NH+]3)N=CC=6CCN5C1C4Sc1c[nH]cn1 | 1 |
| C(=CCCCC=Cc1ccco1)CCC=CCCc1ccoc1 | 3 | C=C1CC23CCC4CCCCC4C2=CCC1C3 | 1 |
| O=C1CC2C(CC3C2CCC2C4CCCCC4CCC23)O1 | 3 | O=C1C=CCCC=CCCCC2CC(CC(C3CSC(=O)N3)O2)O1 | 1 |
| c1ccoc1 | 3 | O=C1CC(=O)c2ccccc2C1=O | 1 |
| C1=CC2(C=CC1)CCNc1cc3[nH]cc4c3c(c12)N=CC4 | 3 | O=C(OC1CCN2CC3CC(CN4C(=O)CCCC34)C2C1)c1ccc[nH]1 | 1 |
| C1=C2CC(OC3CCCCO3)CCC2C2CCC3CCCC3C2C1 | 3 | O=C1CC2C(CCC3Cc4nc5c(nc4CC32)CC2CCC3C4=CC6OC7(CCCCO7)CC6C4CCC3C2C5)C2=CCC3CC4(CCCO4)OCC123 | 1 |
| O=C1C=CC2(C=C1)CC1NCCc3cccc2c31 | 3 | C=C1C=CCCCC=CCC1 | 1 |
| O=C(OC1CCCOC1OC1CCOC(OC2CC3CCC4C5CCC(OC6CCCCO6)CC5=CCC4C3C2)C1)c1ccccc1 | 3 | C1=C2CCCCC2C2CCC3C4CCCC4CCC3C2C1 | 1 |
| O=C1C=CC2C1CC=CC1C3CC3CCC21 | 3 | O=C1OC2CC3(CO3)C(CCc3ccoc3)C3CCCC1C23 | 1 |
| C1=C2CC(OC3CCCCO3)CCC2C2CCC3CC(OC4CCCCO4)CC3C2C1 | 3 | c1cc2c3c(c1)-c1cc4c(cc1CC3NCC2)OCO4 | 1 |
| C1CCC(C2CCCC2)CC1 | 3 | O=C1CCCC2CCC3C4CCC(CCCCCOC5CCCCO5)C4CC(=O)C34CC124 | 1 |
| C1=CC23c4cc5c(cc4COC2CNC3CC1)OCO5 | 3 | O=C(Cc1ccccc1)OC1CC2CCC(C1)N2 | 1 |
| C1CCCCC1 | 3 | O=c1ccc2cc(OC3CCCCO3)ccc2o1 | 1 |
| c1ccc(OC2CCCCO2)cc1 | 3 | O=C1C=C2c3cccc4c(OC5CCCC(COC6CCCCO6)O5)ccc(c34)OC2c2c(OC3CCCCO3)cccc21 | 1 |
| C=C1CCC=CCCC2C=COCC12 | 3 | O=C1CCC=c2cc3ccc4c(c3cc21)OCCC=4 | 1 |
| O=c1c(-c2ccc3c(c2)C=CCO3)coc2ccccc12 | 3 | c1ccc2c(c1)[nH]c1c[nH+]ccc12 | 1 |
| C1=CC23CC[NH+]=c4cc5c6c(c42)N(CCC=6C=N5)C(C1)C3 | 3 | O=C1CCCC2(CO2)C(=O)OC2CCNCC=C(CO1)C2=O | 1 |
| O=C(OC1CCCOC1OC1CCOC(OC2CC3CCC4C5CCC(OC6CCCC(COC7CCC(OC8CCCCO8)CO7)O6)CC5=CCC4C3C2)C1)c1ccccc1 | 3 | C=C1CCC=CCCC2CC(=O)OCC12 | 1 |
| O=C(CCc1ccccc1)c1ccccc1 | 3 | O=C1CCCC23CCCC4(CCCCN4C2)CN13 | 1 |
| O=C(C=Cc1ccccc1)OC1CCCOC1c1cccc2c(=O)ccoc12 | 3 | O=C1CCC2C(C=CC3CC(=O)C(=O)C4CC(=O)CCC34)=CCCC2OC1 | 1 |
| O=C1c2ccccc2OCC12Cc1ccccc12 | 2 | O=C1CCC2C(CCC3=CCCC4OCCCCC34)=CCCC2OC1 | 1 |
| O=C1CCc2ccc3cc[nH]c3c21 | 2 | C1CC2CCC1CO2 | 1 |
| O=C1Cc2ccccc2OC1c1ccccc1 | 2 | c1cc2c3cc1CC1NCCc4ccc5c(c41)Oc1ccc(cc1CO5)CC1NCCc4ccc(c(c41)O2)OC3 | 1 |
| C1CC2CCC34CC35CCCCC5CCC4C2C1 | 2 | C1=C2CC3CCCC2(CC1)C3 | 1 |
| O=c1cc(-c2ccccc2)oc2cccc(C3CC(c4ccccc4)Oc4ccccc43)c12 | 2 | [CH]=c1cc2[nH]c1=Cc1cc3c([nH]1)C(=C1CCC(=N1)C=c1ccc([nH]1)=C2)CC3=O | 1 |
| C1=C2CC(OC3OCCCC3OC3CCCCO3)CCC2C2CCC3C4CCCOC4CC3C2C1 | 2 | c1ccc(C23CCCCC2NCC3)cc1 | 1 |
| O=C1OC2CC1C1C3OC3C3(CO3)C1C2 | 2 | C1CCC2C3=C(CCC2C1)C1CCCC1CC3 | 1 |
| O=C1CCC2C(CCC2C2CCC3(CCCC4OCC(=O)CCC43)O2)OC1 | 2 | C1CCC(OC2CCCC3CCC4C5CC(OC6CCC(OC7CCCO7)CO6)CC5CCC4C32)OC1 | 1 |
| O=C1Cc2ccccc2C2CCCCC12 | 2 | O=C1CC2OC(=O)CC2C(=O)OC2CCN3CC=C(CO1)C23 | 1 |
| C=C1CC=CCCCC2C=COCC12 | 2 | c1c2c(cc3c1OCO3)-c1c3c(cc4c1C(C2)NCC4)OCO3 | 1 |
| C=C1CCCC2CCCCC12 | 2 | O=C1C=CCC2CCC3C4CCC(CCCCCOC5CCCCO5)C4CCC34CC124 | 1 |
| O=C(C=Cc1ccccc1)OC1COC(OCCc2ccccc2)CC1OC1CCCCO1 | 2 | c1cc(C2CCC3C2CCC2C4CCC(OC5CCCC(COC6CCCCO6)O5)CC4CCC23)co1 | 1 |
| O=C(OC1CCOC(OC2CC3CCC4C5CCCCC5=CCC4C3C2)C1)c1ccccc1 | 2 | c1ccc2c(c1)Oc1ccccc1O2 | 1 |
| O=C1C=CCCC1 | 2 | O=C1CCc2ccccc21 | 1 |
| O=c1c(-c2ccccc2)coc2cc3c(cc12)C=CCO3 | 2 | c1ccc(C2CC(c3cccc4c3OC(c3ccccc3)CC4)c3ccccc3O2)cc1 | 1 |
| C1=Nc2cccc3c2c(cn3C2CCCO2)C1 | 2 | O=C(NC1C=CC2CC3=CCC4CCCC4C3CCC2C1)c1ccccc1 | 1 |
| O=C(C=Cc1c[nH]cn1)OC1CC2C=CCCC2C=C(COC2CCCCO2)C2C=CC1O2 | 2 | O=C1C=CCCC=CC=CCCCC2CC(CC(C3CSC(=O)N3)O2)O1 | 1 |
| O=C1OC2CCC(CCc3ccoc3)C3CCCC1C23 | 2 | C1=CCC2CC2C1 | 1 |
| O=C1CCCCCN1 | 2 | C1=CC2CCCC(CCc3ccoc3)C2CC1 | 1 |
| C1=CC2COC=CC2C1 | 2 | c1coc(Cc2cnco2)n1 | 1 |
| C=C1CCC2OCCCCC2C1CCC1CCCC2OCC(=O)CCC12 | 2 | C1CCC(OC2CCCOC2)OC1 | 1 |
| C1=CCC23CCN(Cc4cc5c(cc42)OCO5)C3C1 | 2 | C1=C(C2=NCCCC2)CCCN1 | 1 |
| C=C1C(=O)OC2C1CCC(=C)C1CCC(=C)C12 | 2 | C1CCC2C(C1)CCC1C2CCC2C(C3CCCO3)CCC21 | 1 |
| O=C(CCc1ccccc1)c1cccc(C2CCCCO2)c1 | 2 | O=C1CCC2C(CCC34C=CC(=O)C(=O)C3CC(=O)CC4)=CCCC2OC1 | 1 |
| C=C1CCCCC=CCCCc2coc(c2)C1 | 2 | C1CC(CCC2CCCC34OCC(CCC23)O4)C23CCC(CO2)OC3C1 | 1 |
| O=c1[nH]ccc2c[nH]cc12 | 2 | [CH]=C1C(C2CCCCC2)CCC2COCC12 | 1 |
| O=C1CCCCC(=O)OC2CCNCC=C(CO1)C2=O | 2 | O=C(OCCc1ccccc1)c1ccccc1 | 1 |
| C=c1cccc2c1=CC=C1C2CCC2C1CCC1CC(=O)CCC12 | 2 | O=C1CC2CC(=CC3CCCC4(CCCC(CC(=O)OC5CCOC(C5)CC5CCCC(CCCC=CC6CCCC7(CCCC(C1)O7)O6)O5)O4)O3)CO2 | 1 |
| c1cc2cc(c1)Oc1ccc(cc1)CCc1ccccc1Oc1cccc(c1)CC2 | 2 | c1ccc2c(c1)ncc1cc3c(cc12)OCO3 | 1 |
| c1ccc2c(C3CCCCO3)c3ccccc3cc2c1 | 2 | O=c1ccc2cc3c(cc2o1)OCO3 | 1 |
| O=c1oc2ccccc2c2ccccc12 | 2 | O=C1CCCC2CCC3C4CCC(CCCCCOC5CCCCO5)C4CCC34CC124 | 1 |
| C1=Cc2ccccc2OC1 | 2 | O=c1c2cc3c(cc2c2cccc4c2n1CC4)OCO3 | 1 |
| O=C1CCCC(=O)OC2CCN3CC=C(CO1)C23 | 2 | O=C1CCC2C(CCC34C=CC(C3)C(=O)CC24)C1 | 1 |
| O=C1CCCCC(=O)OC2CCNC=CC(CO1)C2=O | 2 | O=C1CCC2C(CCC3C2CCC2C4CCCCC4CCC23)C1 | 1 |
| O=C1C=C2C3Cc4nc5c(nc4CC3CCC2C23OC2CC2CC4(CCCO4)OCC123)CC1C(CCC2C3=CC4OC6(CCCO6)CC4C3CCC21)C5 | 2 | O=C1c2ccccc2C(C2CCCCO2)c2ccccc21 | 1 |
| C1=C2CCCC(OC3OCCCC3OC3CCCCO3)C2C2CCC3C4CC5(CCCCO5)OC4CC3C2C1 | 2 | O=C(OCC1OC(OC(=O)c2ccccc2)C(OC(=O)c2ccccc2)C(OC(=O)c2ccccc2)C1OC(=O)c1ccccc1)c1ccccc1 | 1 |
| O=c1c(-c2ccccc2)coc2cc(OC3CCCCO3)ccc12 | 2 | O=c1c(-c2ccccc2)coc2c(C3CCCCO3)cccc12 | 1 |
| C1=C(C2CCCCN2)CCCN1 | 2 | C=C1CCC(=O)C2CCCCC12 | 1 |
| O=C(OC1CCC2=CCc3ccccc3C2C1)c1ccccc1 | 2 | O=C1C=CC23CC(Nc4cc5[nH]cc6c5c(c42)N=CC6)SC3C1 | 1 |
| C1CCC2C3=C(CCC2C1)C1CCC2(CCCO2)C1CC3 | 2 | C=C1CCCC(=O)OCC2=CCN3CCC(OC1=O)C23 | 1 |
| O=c1c(Cc2ccccc2)coc2ccccc12 | 2 | O=C(C=Cc1ccccc1)NCCc1c[nH]c2ccccc12 | 1 |
| C1=C2CCCCC2C2CCC3C4CC5(CCCCO5)OC4CC3C2C1 | 2 | c1ccc2c(c1)SSS2 | 1 |
| O=C1CCC2C(CCC3=CC(=O)C(=O)C4CC(=O)CCC34)CCCC2OC1 | 2 | C1=CCCCc2coc(c2)CCC=CCC1 | 1 |
| C=C1CCN2CCCC12 | 2 | O=C1CCCC2C3CC(CN12)C1CCCCN1C3 | 1 |
| C1=C2CCCC(OC3CCCC(COC4CCCCO4)O3)C2C2CCC3CC(OC4CCCCO4)CC3C2C1 | 2 | O=C1CCCC2(CO2)C(=O)OC2CC[NH+]3CC=C(CO1)C23 | 1 |
| C1=C2C(CCC3C2CCC2Cc4nc5c(nc4CC23)CC2CCc3c(cc4c6c3CCC6CC3(CCCO3)O4)C2C5)C2CC3(CCCO3)OC12 | 2 | O=C1C(=O)c2c(ccc3c2CCCC3)C2=C1[CH]CO2 | 1 |
| O=C1CCCCC(=O)OC2CC[NH+]3CC=C(CO1)C23 | 2 | O=C(C=Cc1ccccc1)OC1CCC2C=COC(OC3CCCCO3)C21 | 1 |
| C=C1CC(=O)CO1 | 2 | C1=CCC23c4cc5c(cc4COC2CNC3C1)OCO5 | 1 |
| O=C(C=Cc1ccccc1)OC1CCCOC1c1c(OC2CCCCO2)ccc2c(=O)ccoc12 | 2 | O=C1CCCc2cc3ccccc3cc21 | 1 |
| O=C1CCC2C(CCC34C=CC(CCC23)C4)C1 | 2 | O=c1ccc(C2CCC3C2CCC2C4CCCCC4CCC23)co1 | 1 |
| O=C(OCC1CCCC2C1=CCc1ccccc12)c1ccccc1 | 2 | C1=CC2C3CCCC3CCC23CC32CCCCC12 | 1 |
| O=c1ccoc2ccccc12 | 2 | O=C1CC(=O)C(C(=O)C=Cc2ccccc2)C(=O)C1 | 1 |
| O=C(CCCCCCCCc1ccccc1)c1ccccc1 | 2 | C=C1C=CCCCC(=C)CCC1 | 1 |
| C1=Cc2c[nH]c3cccc(c23)N1 | 2 | O=c1cc(-c2ccccc2)oc2cccc(OC3CCCC(COC4CCCCO4)O3)c12 | 1 |
| O=C1C2OC3(CC(OC4CCCCO4)CCO3)CC2C2CCC3C(CC=C4CCCC(OC5OCC(OC6CCCCO6)CC5OC5CCCCO5)C43)C12 | 2 | C1=C2CC(OC3OCC(OC4CCCCO4)CC3OC3CCCCO3)CCC2C2CCC3C4CC5(CCCCN5)OC4CC3C2C1 | 1 |
| O=C(c1nc(-c2c[nH]c3ccccc23)c[nH]1)c1c[nH]c2ccccc12 | 2 | C=C1C2=CCC(c3ccoc3)C2CC2OC3CC(=O)CCC3C12 | 1 |
| O=C1CC=CCC1 | 2 | C1=C2CCCCC2C2CCC3C(c4ccoc4)CCC3C2C1 | 1 |
| O=C1c2ccccc2C(=O)c2ccccc21 | 2 | O=C(CC(NC(=O)c1ccccc1)c1ccccc1)OC1CC2=CC(=O)C3CCC4OCC4C3C(OC(=O)c3ccccc3)C(C2)C1 | 1 |
| C1=CC2(C=CC1)CC=Nc1cc3[nH]cc4c3c(c12)N=CC4 | 2 | C1CCC2C(C1)CCC1OCCCC12 | 1 |
| O=c1cc(-c2ccccc2)oc2ccc(C3CCCCO3)cc12 | 2 | C=C1CCC2CC1C2 | 1 |
| C1=[NH+]c2c3c(c[nH]c3cc3[nH]cc(-c4ccccc4)c23)C1 | 2 | C1=C2CC(OC3CCC(OC4CCC(OC5CCCCO5)CO4)CO3)CCC2C2CCC3CCCC3C2C1 | 1 |
| C=C1CCCC2CC3OC(=O)C(=C)C3CC12 | 2 | O=C1CCC(=O)C2CC(c3ccc4ccccc4c3)=CCC12 | 1 |
| c1occ2c1CCC1CCCCC21 | 2 | C1=CC23CCC4CCCCC4C2CCC1C3 | 1 |
| O=C1NC(Cc2ccccc2)C(=O)N2CCCC12 | 2 | O=C1CC2C3Cc4nc5c(nc4CC3CCC2C23OC2CC2CC4(CCCO4)OCC123)CC1C(CCC2C3=CC4OC6(CCCO6)CC4C3CCC21)C5 | 1 |
| c1ccc2c(c1)CCC1CCCCC21 | 2 | O=C1C=CCC2N3CCC12c1cc2c(cc1C3)OCO2 | 1 |
| O=C(OC1CC(OC2CCCCO2)COC1OC1CCOC(OC2CC3CCC4C5CCCCC5=CCC4C3C2)C1)c1ccccc1 | 2 | O=C1Cc2c(C3CCCCC3)ccc3c2C(OC3)O1 | 1 |
| C1=Nc2cccc3[nH]cc(c23)C1 | 2 | O=C1C=CC(=O)c2cc(C3=CC(=O)c4ccccc4C3=O)ccc21 | 1 |
| c1ccc(C2CCCC2)cc1 | 2 | C=C1C(=O)OC2C=C3CCC4OC4(CCC12)COC3 | 1 |
| O=C(OCC1CCCC(OC2CCCC3CCC4C5CC(OC6CCCCO6)CC5CCC4C32)O1)c1ccccc1 | 2 | O=C1CC=CC2Cc3ccoc3CC12 | 1 |
| C1=C2CCCC(OC3OCCC(OC4CCCCO4)C3OC3CCCCO3)C2C2CCC3C4CC5(CCCCO5)OC4CC3C2C1 | 2 | O=c1c2c(c3[nH]ccc4cnc1c43)C(c1ccccc1)CN2 | 1 |
| C=C1CCC2OC2CCC2C=COCC12 | 2 | C=C1C2=CCC(c3ccoc3)C2CCC1C1CCC(=O)CC1 | 1 |
| O=C(OCC1CCCC(Oc2c(-c3ccccc3)oc3ccccc3c2=O)O1)c1ccccc1 | 2 | C1=CC(CCCC2C=CCOO2)OOC1 | 1 |
| O=C1CCC2C(=CCC3C4CCCC4CCC23)C1 | 2 | O=C1CC2(CCCO2)C2CCC3=C(CCC4CCCCC34)C12 | 1 |
| c1ccc2c(c1)SSSSS2 | 2 | C=C1C(=O)OC2CCCCC12 | 1 |
| O=C1OC2CCC3CCC(CC=CC=CCCC4CCCC(CCC1C2)O4)O3 | 2 | C1CCNC1 | 1 |
| O=C(OC1CCCOC1OC1CCOC(OC2CC3CCC4C5CCCCC5=CCC4C3C2)C1)c1ccccc1 | 2 | O=C1c2ccccc2OC2Oc3cc4c(cc3C12)C=CCO4 | 1 |
| O=C(OC1CCCOC1OC1CCOC(OC2CC3CCC4C5CCC(OC6CCCC(COC7CCCCO7)O6)CC5=CCC4C3C2)C1)c1ccccc1 | 2 | C1=C2CC(OC3OCCCC3OC3CCCCO3)CCC2C2CCC3C(c4ccoc4)CCC3C2C1 | 1 |
| O=C1C=CC2C(CCC3C4CCCC4CCC23)C1 | 2 | C1=C2CCN3Cc4ccccc4C(CC1)C23 | 1 |
| c1cc2c3c(c1)Oc1ccc(cc1)CC1NCCc4ccc5c(c41)Oc1ccc(cc1CO5)CC3NCC2 | 2 | O=C1C=CC2CC(OC(=O)Cc3ccccc3)C3C=CC(C=CC2C1)O3 | 1 |
| O=C1C=C2c3cccc4c(OC5CCCC(COC6CCCCO6)O5)ccc(c34)OC2c2c(OC3CCCC(COC4CCCCO4)O3)cccc21 | 1 | c1ccc2c(c1)CCCC2 | 1 |
| [CH]=C1C(=O)CC2C1CCC13CC14CCCCC4CCC23 | 1 | C1=CC2C(CC1)CCC1C3CCCC3CCC21 | 1 |
| C=C1C(=O)OCC2CCCCC12 | 1 | C=c1ccc2cc(=c3c4c(ccc3=C)C(=O)C=CC4=O)ccc2c1 | 1 |
| C=C1CCCC(=O)CC2CCCC3(CCCC(C=CCCCC4CCCC(CC5CC(CCO5)OC(=O)CC5CCCC6(CCCC(C1)O6)O5)O4)O3)O2 | 1 | O=c1c2c(c3c4cccc5c(=O)c6c(c(c7cccc1c73)c54)CCOC6)CCOC2 | 1 |
| c1ccc(CCCC23CCC(CCO2)O3)cc1 | 1 | O=C1CC2CCC=CCCC=CC2O1 | 1 |
| O=C1OC2CNC3CCC=CC32c2cc3c(cc21)OCO3 | 1 | C1=C2CCCCC2C2CCC3C4CCCCC4CCC3C2C1 | 1 |
| O=C1CC=C2CCC3C=CC(C=CC2C1)O3 | 1 | c1ccc(Cc2ccoc2)cc1 | 1 |
| O=C1CC2C3CCCC=C3CCC2C2CCC(c3ccc(=O)oc3)C12 | 1 | O=C1CC2C(CCC3Cc4nc5c(nc4CC32)CC2CCC3C4=CC6OC7(CCCO7)CC6C4CCC3C2C5)C2=CCC3CCOCC123 | 1 |
| C1=CCCC2CC2C=CCC1 | 1 | O=C(C=Cc1ccccc1)OC1CCCCC1 | 1 |
| O=c1ccc(C2CCC3C2CCC2C4CCC(OC5CC(OC6CCCCO6)C(OC6CCCCO6)CO5)C=C4CCC23)co1 | 1 | C1=CC2C3CCCC3CCC2C2CCCCC12 | 1 |
| O=C1CCCC(CCC2CCCC3OC4CCC23OC4)C1 | 1 | c1ccc2c(Cc3nc(-c4c[nH]c5ccccc45)c[nH]3)c[nH]c2c1 | 1 |
| O=c1ccc(C2CCC3C2CCC2C4CCC(OC5CCCCO5)CC4CCC23)co1 | 1 | O=c1c(OC2CCCO2)c(-c2ccccc2)oc2ccccc12 | 1 |
| c1cc2cc3cc(OCC4CO4)ccc3nc2o1 | 1 | C=C1C(=O)OC2C1CCC1COC(=O)C(=C)C12 | 1 |
| O=C1NCC(c2c[nH]c3ccccc23)NC1c1c[nH]c2ccccc12 | 1 | O=C1CCC2C(=O)C(C3CC=CC3)CC(=O)C2C1 | 1 |
| O=C(NC1C=CC2CC3=CCC4CCCC4C3CC3OCC1C23)c1ccccc1 | 1 | c1cc2cc(c1)Oc1ccc3c(c1)C(Cc1ccc(cc1)Oc1cccc4c1C(C2)NCC4)NCC3 | 1 |
| O=C(C=Cc1ccccc1)OCCc1ccccc1 | 1 | O=C1C=C(CCC2CCCC3OCC(=O)CCC23)C2CCC(=O)CC2C1 | 1 |
| O=C(OC1CC2COC3(C=CC(=O)C(OC(=O)c4ccoc4)C13)C2)c1ccoc1 | 1 | C(=Cc1ccc2c(c1)OCO2)c1ccccc1 | 1 |
| O=C1CC=CC2C=c3ccoc3=CC12 | 1 | O=C1CCC2(c3ccccc3)CCNC2C1 | 1 |
| O=C1CCC23C(=O)CCCC4OOC(C=C12)CC43 | 1 | C1=CCCC=CCCC=CC1 | 1 |
| O=C1C=C2C3OC34CCC3CCCCC3C4C3OC23O1 | 1 | C(=Cc1cccc(OC2CCCCO2)c1)c1ccccc1 | 1 |
| O=C1OC2CC3CCCCC13c1ccccc12 | 1 | O=C(OC1OC2COC(=O)c3ccccc3-c3ccccc3C(=O)OC2C(OC(=O)c2ccccc2)C1OC(=O)c1ccccc1)c1ccccc1 | 1 |
| C1=C2COCC2C2CCCCC2C1 | 1 | C1=CC23CCN(Cc4ccccc42)C3CC1 | 1 |
| C=C1C(=O)OC2=CC=C3CC(=O)C(CCC12)O3 | 1 | c1cc(C2CCC3C2CCC2C4CCCCC4CCC23)co1 | 1 |
| O=C1CC=C2C3CCCCC123 | 1 | O=C1C2OC3(CCCCO3)CC2C2CCC3C(CC=C4CCCC(OC5OCCCC5OC5CCCCO5)C43)C12 | 1 |
| O=C(C=Cc1ccccc1)OC1CCC2C(CCC3C2CCC2C4CCCC4CCC23)C1 | 1 | C1=CC2CC=CCC2CCC1 | 1 |
| O=c1cc(-c2ccccc2)oc2cccc(OC3CCCCO3)c12 | 1 | O=C1C=C(C2=CC(=O)c3ccccc3C2=O)C(=O)c2ccccc21 | 1 |
| C1=CCc2ccoc2CC=CCC1 | 1 | C=C1C(=O)OC2C1CCCC1CC=CC12 | 1 |
| C=C1CC[NH+]2CCCC12 | 1 | C1CCC2CCCC2CC1 | 1 |
| C1=C2C(CCC3CCCC4OCC5CCC34O5)CCCC2OCCC1 | 1 | [CH]=c1ccc2cc3c(cc2c1)C(=O)CCC=3 | 1 |
| O=C1CCCCC1=O | 1 | O=C1CCc2cccc(c2)Oc2ccc(cc2)C2OC2CO1 | 1 |
| C1=C2C3CC4CC5N(CCC35c3ccccc3)C2(CC2NCCC12c1ccccc1)O4 | 1 | O=C1CCCC2(CO2)C(=O)OC2CCNC=CC(CO1)C2=O | 1 |
| C=C1CCCC2CCCC2C1 | 1 | C1=C2CC(OC3OCCC(OC4CCCCO4)C3OC3CCCCO3)CCC2C2CCC3C4CC5(CCCCN5)OC4CC3C2C1 | 1 |
| C=C1CCCC(=O)OCC2=CC[NH+]3CCC(OC1=O)C23 | 1 | O=C(CCCC(=O)OC1CCOCC1)Cc1cc(=O)cco1 | 1 |
| [CH]=C1CCCC(=O)OCC2CCN3CCC(OC1=O)C23 | 1 | C1=C2CC34CCCCC3CC(O4)C2C2CCC(CCCCCOC3CCCCO3)C2C1 | 1 |
| O=C(NC1C=CC23CC4=CCC5CCCC5C4CCC2C1CO3)c1ccccc1 | 1 | O=C1CC(CCc2ccoc2)C2CCCCC2C1=O | 1 |
| c1ccc2c(c1)CCc1ccoc1C2 | 1 | C=C1CCCC2C1C=CC1C(=O)OCC12 | 1 |
| O=C1CC(=O)C(C(=O)CCc2ccccc2)C(=O)C1 | 1 | C1CCC2(CC3C(CC4C3CCC3C5CCCCC5CCC34)O2)OC1 | 1 |
| [CH]=C1CCCC(=O)OCC2=CCN3CC(CC23)OC1=O | 1 | [CH]=C1C(CC(=O)OCCc2ccccc2)C=COC1OC1CCCCO1 | 1 |
| O=C(OC1CCCC23CC(CO2)CC(OC(=O)c2ccoc2)C13)c1ccoc1 | 1 | C1=CC2CCc3ccoc3C(C1)C2 | 1 |
| C=C1CCCCCCCC(=O)CCCC(=O)C1=O | 1 | c1ccc(C2CNCc3ccccc32)cc1 | 1 |
| O=C(OC1CCOC(OC2CCOC(OC3CC4CCC5C6CCC(OC7CCCC(COC8CCC(OC9CCCCO9)CO8)O7)CC6=CCC5C4C3)C2)C1)c1ccccc1 | 1 | C1=CC2CCCC=C2CC1 | 1 |
| O=C(C=Cc1ccccc1)OC1CCCOC1OC1CCOC(OC2CC3CCC4C5CCC(OC6CCCCO6)CC5=CCC4C3C2)C1 | 1 | O=c1ccc2c3cccc4c(=O)c5c(c(c6c7c(cc1c26)COCC7)c43)CCOC5 | 1 |
| O=C1CC2CCCC3CCC4OOC32C(O1)O4 | 1 | O=C(OC1CCC(OC2CCCOC2OC2CCC3C(=CCC4C3CCC35COC6CCOC(CC43)C65)C2)OC1)c1ccccc1 | 1 |
| C=C1CCCC=CCC2C(=C)C(=O)OC2C1 | 1 | O=C1OC2CCC3CCC(CC=CC=CCCC4CC(OC5CCCCO5)CC(CCC1C2)O4)O3 | 1 |
| C(=CC=CC=CC=CC=CC1C=CCCC1)C=CC=CC=CC=CC1=CCCCC1 | 1 | O=c1cc(-c2cccc(-c3cccc4c(=O)cc(-c5ccccc5)oc34)c2)oc2ccccc12 | 1 |
| C=C1C2=CCC(c3ccoc3)C2CCC1C1CCCCC1 | 1 | O=C1CC2CCCCC2C(CCc2ccoc2)C1 | 1 |
| C=C(Cc1ccccc1)C(=C)Cc1ccccc1 | 1 | C(#Cc1ccc(OC2CCCCO2)cc1)CC=Cc1ccc(OC2CCCCO2)cc1 | 1 |
| O=C1CCC2C(CCC3CCCC4OC5CCC34OC5)=CCCC2OC1 | 1 | C1CCC2(CC3C(CC4=C5CCC6CCCCC6C5CCC43)O2)OC1 | 1 |
| O=C1CCC2(O1)C(=O)OC1CC3C4CCC5CCCCC5C4CCC3C12 | 1 | [CH]=C1CCCC(=O)OCC2=CCNCCC(OC1=O)C2=O | 1 |
| C1=CCC=CCc2cc(co2)CCCC=C1 | 1 | O=C1C=C2CC3CCCC2(C1)C3 | 1 |
| c1ccc2ccccc2c1 | 1 | O=C1NCCc2cn(C3CCCO3)cc21 | 1 |
| C1=C2CC(OC3OCCCC3OC3OCCCC3OC3CCCCO3)CCC2C2CCC34COC5CCOC(CC3C2C1)C54 | 1 | O=C1CCc2cccc(c2)Oc2ccc(cc2)C=CCO1 | 1 |
| C=C1CCC2OC2CCC2CCC12 | 1 | C1CCC(OC2CCCC3CCC4C5CC6OC7(CCCCO7)CC6C5CCC4C32)OC1 | 1 |
| O=C1CC2CCCCC2c2ccccc21 | 1 | O=C1CCC2C(CCc3ccoc3)CCCC2C1 | 1 |
| C(Cc1ccccc1)=Nc1cc2c3c(c[nH]c3c1)CC=N2 | 1 | O=C(Cc1ccccc1)OC1CC2NC(C1)C1OC21 | 1 |
| O=c1ccc2cc(CC3CO3)ccc2o1 | 1 | O=C1CCCCC2OC2CCC(=O)C(=O)CCC1 | 1 |
| C1CCC2CC(CO2)OC1 | 1 | C=C1CCCC2CCCC2C1=O | 1 |
| C1=C2CC(OC3OCCCC3OC3OCCCC3OC3CCCCO3)CCC2C2CCC3C4CCCOC4CC3C2C1 | 1 | O=C1C(=O)C2CCCCC2c2ccccc21 | 1 |
| O=C1c2ccccc2OCC12Cc1cc3c(cc12)OCO3 | 1 | O=C1CC2CCC34COCC(=CC2O1)CCC3O4 | 1 |
| C1=C2C3CCCCC3CCC2C2CC3OC4(CCCCO4)CC3C2C1 | 1 | O=C1c2cc3c(cc2OC2Oc4cc5c(cc4C12)C=CCO5)OCC=C3 | 1 |
| O=C1CCCC2(CO2)C(=O)OC2CCN3CC=C(CO1)C23 | 1 | O=C1Oc2ccccc2C12CCCCC2 | 1 |
| C1CCCCNCCCNCCCC1 | 1 | c1ccc2c(c1)[SH+]c1ccccc1[SH+]2 | 1 |
| O=c1ccc2c3c(ccc2o1)OCC=C3 | 1 | O=C(C=CC=CC=CC=CC=CC=CC=CC=C=C1CCCCC1)CC12CCCCC1O2 | 1 |
| O=C1CCOC2CCC3CCCCC3C12 | 1 | C1=C2C(CCC3C2CC2OC4(CCCCO4)CC23)C2CCCCC2C1 | 1 |
| C1CC2CCC[NH+]2C1 | 1 | c1ccc(CCNCc2ccccc2)cc1 | 1 |
| O=c1c(-c2ccc3c(c2)C=CCO3)coc2cc3c(cc12)C=CCO3 | 1 | c1ccc2c(c1)OCO2 | 1 |
| C1CCC2C(C1)OC1CCCC12 | 1 | O=c1ccc(C2CCC3C2CCC2C4CCCC=C4CCC23)co1 | 1 |
| c1cc2ccc3c(c2[nH]1)CCC3 | 1 | C1=CC2CC(C1)C2 | 1 |
| C=C1CCC=CC1=O | 1 | [CH]=c1cc2ccccc2cc1=C | 1 |
| C1CC2CCCC2C2CC2C1 | 1 | O=c1c(-c2ccccc2)coc2cc3c(cc12)CCO3 | 1 |
| O=c1cc(-c2ccccc2)oc2c(C3CCCCO3)cc(C3CCCCO3)cc12 | 1 | O=C(OCC1CC(OC(=O)c2ccccc2)C(OC(=O)c2ccccc2)C(OC(=O)c2ccccc2)O1)c1ccccc1 | 1 |
| O=C1C=C2CC3C=CCC(=O)C3CC2O1 | 1 | O=C1CCC2C(CCC3=CC=CC4CCCCC34)CCCC2OC1 | 1 |
| O=C1CCC2C(CCC3C4CCC5CCCCC5C4=CCC23)C1 | 1 | C1=C2CC(OC3CCCC(COC4CCC(OC5CCCCO5)CO4)O3)CCC2C2CCC3CC(OC4CC(OC5CCCCO5)CCO4)CC3C2C1 | 1 |
| O=c1ccoc2c(C3CCCCO3)cccc12 | 1 | O=C1CCCc2ccccc21 | 1 |
| O=C1C=CC23CC(Nc4cc5[nH]cc6c5c(c42)N=CC6)SC3=C1 | 1 | C=C1CC23CCC4CCCCC4C2CCC1C3 | 1 |
| O=C1CCCCN1 | 1 | C1=C2C3CCCCC3CCC2C2CCC3CC(OC4CC(OC5CCCCO5)CCO4)CCC3C2C1 | 1 |
| C1CCC(C2=NCCC2)NC1 | 1 | C1=CC23CCNCc4cccc(c42)OC3CC1 | 1 |
| C1=C2C(CCC3C2CCC2Cc4nc5c(nc4CC23)CC2CCC3C4=CC6OC7(CCCO7)CC6C4CCC3C2C5)C2CC3(CCCCO3)OC12 | 1 | O=C1CCC2OC2CC2CC(CC=CC=CCc3cccc(c3)N1)NC(=O)O2 | 1 |
| [CH]=C1CCCC(=O)OCC2CC[NH+]3CCC(OC1=O)C23 | 1 | c1ccc(CC2NCCc3ccccc32)cc1 | 1 |
| C=C1C=CCCC1 | 1 | O=C1C=CC23CCNC(=C2C1)Cc1ccccc13 | 1 |
| C1CCC2(CC1)CC1OC1C1OC12 | 1 | O=C1NC2CCC=CC23c2cc4c(cc2CN(CCc2ccccc2)C13)OCO4 | 1 |
| C=C1CC2OC(=O)C(=C)C2CCC2OC1=CC2=O | 1 | C1=C2C3CCCC3CCC2C2CCCCC2C1 | 1 |
| O=C1C=CCCCC(=O)OCC2=CCN3CCC(O1)C23 | 1 | O=C(C=Cc1ccccc1)OCC1CCCC(Oc2c(-c3ccccc3)oc3ccccc3c2=O)O1 | 1 |
| C1=CCOOC1 | 1 | O=C1C=CC(=O)c2cc3cnccc3cc21 | 1 |
| C1=C2CC(OC3CCCC(COC4CCCCO4)O3)CCC2C2CCC3CC(OC4CC(OC5CCCCO5)CCO4)CC3C2C1 | 1 | O=C1CC=C2C1CC=CC1C2CCC2CC21 | 1 |
| [CH]=C1CCCC(=O)OCC2CCNCCC(OC1=O)C2=O | 1 | C1CCC2CCCCC2C1 | 1 |
| O=c1ccc2cc3c(cc2o1)OCC3 | 1 | c1ccc(CCOc2ccccc2)cc1 | 1 |
| C=C1C(=O)C23CCC4C5CCCC4(OC5=O)C2CCC1C3 | 1 | O=C1CC=CC1 | 1 |
| O=C(c1ccccc1)c1ccccc1 | 1 | O=c1c(OC2CCCC(COC3CCCCO3)O2)c(-c2ccccc2)oc2ccccc12 | 1 |
| c1c2c(cc3c1OCO3)C1CCCC3CCN(C2)C31 | 1 | c1ccc2c(c1)NCc1cc3c(cc1-2)OCO3 | 1 |
| C=C1CCC2C3CCCCC3CCC2C1CCc1ccoc1 | 1 | O=C1CC2OCC2C(=O)OC2CCN3CC=C(CO1)C23 | 1 |
| O=C1c2ccoc2CCC2CCCC12 | 1 | O=C1C2OC3(CC(OC4CCCCO4)CCO3)CC2C2CCC3C(CC=C4CCCC(OC5OCCCC5OC5CCCCO5)C43)C12 | 1 |
| O=C1CC2CCCCC2c2cocc21 | 1 | C1=CC2CCC1CCc1ccoc12 | 1 |
| O=C1CC2C(c3ccc(=O)oc3)CCC2C2CCC3=CC4OC5CCOC(C5)OC4CC3C12 | 1 | O=C1C=CC2(C=C1)CCNc1cc3[nH]cc4c3c(c12)N=CC4 | 1 |
| C1=C2CCCC3c4ccccc4CN(C1)C23 | 1 | O=c1ccc2cc3c(=O)cc4c(c3oc2c1)=CCCO4 | 1 |
| O=C1c2occc2CC2CCCCC12 | 1 | C=c1cc2ccccc2cc1=c1c2c(ccc1=C)C(=O)C=CC2=O | 1 |
| C1=Nc2c3c(c[nH]c3cc3[nH]cc(-c4ccccc4)c23)C1 | 1 | O=C(C=Cc1ccccc1)OC1CCCOC1OC1CCOC(OC2CC3CCC4C5CCCCC5=CCC4C3C2)C1 | 1 |
| C1=C2CCCCC2C2CCC3CC(OC4CC(OC5CCCCO5)CCO4)CC3C2C1 | 1 | c1ccc(C2OCC3C(c4ccccc4)OCC23)cc1 | 1 |
| O=C(OC1CC2COC3(CCC(=O)C(OC(=O)c4ccoc4)C13)C2)c1ccoc1 | 1 | c1c2c(cc3c1OCO3)C13CCCCC1N(CC3)C2 | 1 |
